# Toxicokinetics and toxicodynamics of the fentanyl homologs cyclopropanoyl-1-benzyl-4´-fluoro-4-anilinopiperidine and furanoyl-1-benzyl-4-anilinopiperidine

**DOI:** 10.1007/s00204-020-02726-1

**Published:** 2020-04-05

**Authors:** Tanja M. Gampfer, Lea Wagmann, Yu Mi Park, Annelies Cannaert, Jennifer Herrmann, Svenja Fischmann, Folker Westphal, Rolf Müller, Christophe P. Stove, Markus R. Meyer

**Affiliations:** 1 Department of Experimental and Clinical Toxicology, Institute of Experimental and Clinical Pharmacology and Toxicology, Center for Molecular Signaling (PZMS), Saarland University, 66421 Homburg, Germany; 2 Department of Microbial Natural Products (MINS), Helmholtz Institute for Pharmaceutical Research Saarland (HIPS), Saarland University, 66123 Saarbrücken, Germany; 3 Laboratory of Toxicology, Department of Bioanalysis, Faculty of Pharmaceutical Sciences, Ghent University, 9000 Ghent, Belgium; 4 State Bureau of Criminal Investigation Schleswig-Holstein, 24116 Kiel, Germany; 5 Environmental Safety Group, Korea Institute of Science and Technology (KIST) Europe, 66123 Saarbrücken, Germany

**Keywords:** In vitro and in vivo metabolism, Metabolic stability, LC–HRMS/MS, Zebrafish larvae, In vitro µ-opioid receptor activity

## Abstract

The two fentanyl homologs cyclopropanoyl-1-benzyl-4´-fluoro-4-anilinopiperidine (4F-Cy-BAP) and furanoyl-1-benzyl-4-anilinopiperidine (Fu-BAP) have recently been seized as new psychoactive substances (NPS) on the drugs of abuse market. As their toxicokinetic and toxicodynamic characteristics are completely unknown, this study focused on elucidating their in vitro metabolic stability in pooled human liver S9 fraction (pHLS9), their qualitative in vitro (pHLS9), and in vivo (zebrafish larvae) metabolism, and their in vitro isozyme mapping using recombinant expressed isoenzymes. Their maximum-tolerated concentration (MTC) in zebrafish larvae was studied from 0.01 to 100 µM. Their µ-opioid receptor (MOR) activity was analyzed in engineered human embryonic kidney (HEK) 293 T cells. In total, seven phase I and one phase II metabolites of 4F-Cy-BAP and 15 phase I and four phase II metabolites of Fu-BAP were tentatively identified by means of liquid chromatography high-resolution tandem mass spectrometry, with the majority detected in zebrafish larvae. *N*-Dealkylation, *N*-deacylation, hydroxylation, and *N*-oxidation were the most abundant metabolic reactions and the corresponding metabolites are expected to be promising analytical targets for toxicological analysis. Isozyme mapping revealed the main involvement of CYP3A4 in the phase I metabolism of 4F-Cy-BAP and in terms of Fu-BAP additionally CYP2D6. Therefore, drug-drug interactions by CYP3A4 inhibition may cause elevated drug levels and unwanted adverse effects. MTC experiments revealed malformations and changes in the behavior of larvae after exposure to 100 µM Fu-BAP. Both substances were only able to produce a weak activation of MOR and although toxic effects based on MOR activation seem unlikely, activity at other receptors cannot be excluded.

**Electronic supplementary material:**

The online version of this article (10.1007/s00204-020-02726-1) contains supplementary material, which is available to authorized users.

## Introduction

More and more compounds intended to be consumed as substitutes and/or alternatives to classic opioids such as heroin are brought into the drugs of abuse market (Beardsley and Zhang 2018). They are summarized under the term new synthetic opioids (NSO) and have markedly contributed to the dramatic rise in overdose deaths amongst opioid abusers (Fagiola et al. 2018; Guerrieri et al. 2017; Muller et al. 2019; Sharma et al. 2019; Solimini et al. 2018). This is partly due to their nM affinity at the µ-opioid receptor (MOR) and their enhanced brain penetration owing to higher lipophilicity, but the influence of other toxicodynamic effects cannot be excluded as they were often not characterized prior to abuse (Baumann et al. 2018). Limited data are also available concerning their toxicokinetics, which is important amongst other factors in forensic and clinical toxicology and doping control for developing analytical procedures to detect these compounds in human biosamples (Wagmann and Maurer 2018). Furthermore, the knowledge about the toxicokinetics and toxicodynamics of emerging NSO and other drugs of abuse is essential for law enforcement personnel and policymakers to allow thorough risk assessment (Evans-Brown and Sedefov 2018).

**Fig. 1:**
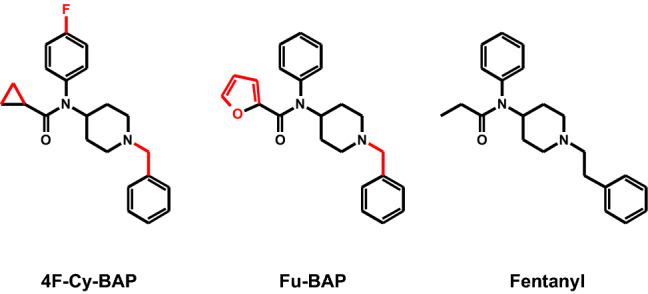
Chemical structures of cyclopropanoyl-1-benzyl-4´-fluoro-4-anilinopiperidine (4F-Cy-BAP), furanoyl-1-benzyl-4-anilinopiperidine (Fu-BAP), and fentanyl. Structural deviations from fentanyl are highlighted in red (online version only)

The two fentanyl homologs cyclopropanoyl-1-benzyl-4′-fluoro-4-anilinopiperidine (4F-Cy-BAP) and furanoyl-1-benzyl-4-anilinopiperidine (Fu-BAP) have been seized in Europe and were intended to be brought onto the market as NSO (EMCDDA 2018). Their chemical structures, in comparison to fentanyl, are given in Fig. 1. Fu-BAP is structurally related to furanylfentanyl, which was risk assessed by the EMCDDA in 2017 (EMCDDA 2017). Furanylfentanyl differs from Fu-BAP by replacement of the phenylethylamine part with phenylmethylamine. So far, nothing is known about the toxicokinetic and toxicodynamic characteristics of 4F-Cy-BAP and Fu-BAP. However, *N*-(1-benzylpiperidin-4-yl)-arylacetamides, structurally related compounds, were described to be potent agonists at the sigma receptor (Huang et al. 2001), with an affinity of *N*-(1-benzylpiperidin-4-yl)-arylacetamide at the sigma1 and sigma2 receptor of 3.9 and 240 nM, respectively. Fu-BAP and related compounds were also identified as antagonists at the acetylcholine M2 and M3 receptor with Ki values of 794 and 100 nM for Fu-BAP, respectively (Diouf et al. 2002).

To close the knowledge gap concerning their toxicokinetics and toxicodynamics, the present study aimed to elucidate the toxicokinetics of these compounds, including in vitro metabolism in pooled human liver S9 fraction (pHLS9) incubations in comparison to in vivo metabolites identified using the zebrafish larvae model, isozyme mapping, and the determination of plasma protein binding (PPB). Toxicodynamic properties should include characterizing the MOR activity in engineered human embryonic kidney (HEK) 293 T cells as well as maximum-tolerated concentration (MTC) studies in zebrafish larvae.

## Materials and methods

### Chemicals and reagents

4F-Cy-BAP and Fu-BAP were provided as citrate salts for research purposes from the EU-project ADEBAR/State Bureau of Criminal Investigation Schleswig–Holstein (Kiel, Germany). Chemical purity and identity of the compounds were verified by mass spectrometry (MS) and nuclear magnetic resonance analysis. Stock solutions in methanol (1 mg/mL) or DMSO were freshly prepared before each experiment. Hydromorphone was purchased as hydromorphone HCl from Fagron (Nazareth, Belgium). Fentanyl was obtained as a free base from LGC Chemicals (Wesel, Germany). Trimipramin-d_3_, isocitrate, isocitrate dehydrogenase, superoxide dismutase, 3′-phosphoadenosine-5′phosphosulfate (PAPS), S-(5′-adenosyl)-l-methionine (SAM), dithiothreitol (DTT), reduced glutathione (GSH), magnesium chloride (MgCl_2_), potassium dihydrogen phosphate (KH_2_PO_4_), dipotassium hydrogen phosphate (K_2_HPO_4_), tris hydrochloride, fetal bovine serum (FBS), and poly-d-lysin were from Sigma Aldrich (Taufkirchen, Germany/Overijse, Belgium) and NADP + from Biomol (Hamburg, Germany). Centrifree devices were obtained from Merck (Darmstadt, Germany). Dulbecco’s Modified Eagle’s Medium (DMEM; GlutaMAX™), Opti-MEM® I Reduced Serum Medium, penicillin–streptomycin (5.000 U/mL) and amphotericin B (250 µg/mL) were purchased from Thermo Fisher Scientific (Pittsburg, PA, USA). The Nano-Glo® Live Cell reagent, which was used for the readout of the MOR bioassay, was procured from Promega (Madison, WI, USA). Acetonitrile (LC–MS grade), methanol (LC–MS grade), ammonium formate (analytical grade), formic acid (LC–MS grade), and all other reagents and chemicals (analytical grade) were from VWR (Darmstadt, Germany). Zebrafish embryos were obtained from in-house bred adult zebrafish of the AB wild-type line. The baculovirus-infected insect cell microsomes (Supersomes) containing human cDNA-expressed flavin-containing monooxygenase 3 (FMO3) (5 mg protein/mL), CYP1A2, CYP2A6, CYP2B6, CYP2C8, CYP2C19, CYP2D6, CYP3A4 (1 nmol/mL), CYP2C9, CYP2E1, or CYP3A5 (2 nmol/mL), as well as pooled human liver microsomes (pHLM, 20 mg microsomal protein/mL, 330 pmol total CYP/mg protein), pooled human liver S9 fraction (pHLS9; 20 mg microsomal protein/mL), UGT reaction mixture solution A (25 mM UDP-glucuronic acid), and UGT reaction mixture solution B (250 mM Tris HCl, 40 mM MgCl2, and 125 μg/mL alamethicin) were supplied by Corning (Amsterdam, The Netherlands). After delivery, the enzymes were thawed at 37 °C, aliquoted, snap-frozen in liquid nitrogen, and stored at − 80 °C until use.

### In vitro metabolic stability, identification of in vitro metabolites, and plasma protein binding

According to a previous study (Gampfer et al. 2019), pHLS9 (2 mg microsomal protein/mL) was preincubated for 10 min at 37 °C with 25 μg/mL alamethicin (UGT reaction mixture solution B), 90 mM phosphate buffer (pH 7.4), 2.5 mM Mg^2+^, 2.5 mM isocitrate, 0.6 mM NADP + , 0.8 U/mL isocitrate dehydrogenase, 100 U/mL superoxide dismutase. Thereafter, 2.5 mM UDP-glucuronic acid (UGT reaction mixture solution A), 40 μM PAPS, 1.2 mM SAM, 1 mM DTT, 10 mM GSH was added. To ensure a linear metabolism during incubation, the compound concentrations were set at 2.5 µM (Baranczewski et al. 2006). The given concentrations are concentrations in the final incubation mixtures (300 µL final volume). All incubations were done in duplicate. The organic solvent content was kept below 1% (*v/v*) (Chauret et al. 1998).

Reactions were initiated after addition of 4F-Cy-BAP or Fu-BAP and continued for 360 min. Meanwhile, 30 µL samples were taken after 1, 15, 30, 45, 60, 75, 90, 180, and 360 min, respectively. Reactions were stopped by adding 10 µL ice-cold acetonitrile. Afterwards, the samples were cooled for 30 min at − 20 °C, centrifuged at 18,407×*g* for 2 min, and the supernatants were transferred to autosampler vials, and measured by liquid chromatography high-resolution tandem MS (LC–HRMS/MS). In order to identify metabolites formed by NADP^+^ independent enzymes, incubations without NADP^+^ were also performed. Blank incubations without substrate and control incubations without enzyme (pHLS9) were prepared to examine whether interfering or non-metabolically formed compounds were present.

Metabolic stability was determined by declining substrate concentration (Wagmann et al. 2019), plotting the natural logarithm of the absolute peak area ratios of 4F-Cy-BAP or Fu-BAP versus time, respectively. In vitro half-lives were calculated by the slope of the respective linear regression. A *t*-test was done to confirm that there was no significant difference between the compound concentration at 360 min in control incubations and the initial concentrations in the pHLS9 incubations at 1 min. GraphPad Prism 5.00 (GraphPad Software, San Diego, USA) was used for statistical calculations with the following defined settings: unpaired; two-tailed; significance level, 0.05; confidence intervals, 99%.

In vitro half-life (*t*_1/2_) and intrinsic clearance (CL_int,_ Eq. 1–3) were determined in accordance to Baranczewski et al. (2006). Hepatic clearance (CL_h_) was predicted using parallel tube model with (Eq. 4) and without (Eq. 5) free fraction in plasma (*f*_u_) and well-stirred model with (Eq. 6) and without (Eq. 7) *f*_u_ (Obach 1999). Calculations of hepatic extraction ratio (ER_h_, Eq. 8) were based on Eqs. 5 and 7 (Mehvar 2018).

1 $$ t_{1/2} ,{\min} = \frac{\ln 2 }{{k }} $$

2 $$ {\ln}\left[ {\text{peak area ratio}} \right]_{{\text{remaining }}} = {\ln}\left[ {\text{peak area ratio}} \right]_{{{\text{initial}}}} - k \times t $$

3 $$ {\text{CL}}_{{{\text{int}}}} ,{\text{mL}}/{\min}/{\text{kg}} = \frac{\ln 2}{{t_{1/2} }} \times \frac{{\left[ {\text{V}} \right]_{{{\text{incubation}}}} { }}}{{\left[ {\text{P}} \right]_{{{\text{incubation}}}} }} \times \frac{{\left[ {{\text{Liver}}} \right]{ }}}{{\left[ {{\text{BW}}} \right]{ }}} \times {\text{SF}} $$

4 $$ {\text{CL}}_{{\text{h}}} ,{\text{mL}}/{\min}/{\text{kg}} = {{ Q}} \times \left( {1 - {\text{e}}^{{\left( {{ }\frac{{ - f_{u } \times {\text{ CL}}_{{{\text{int}}}} }}{{\text{Q}}}} \right)}} } \right) $$

5 $$ {\text{CL}}_{{\text{h}}} ,{\text{mL}}/{\min}/{\text{kg}} = {{ Q}} \times \left( {1 - {\text{e}}^{{\left( {{ }\frac{{{ } - {\text{ CL}}_{{{\text{int}}}} }}{{\text{Q}}}} \right)}} } \right) $$

6 $$ {\text{CL}}_{{\text{h}}} ,{\text{mL}}/{\min}/{\text{kg}} = { }\frac{{{{Q}} \times f_{u } \times {\text{CL}}_{{{\text{int}}}} { }}}{{{{Q}} + f_{{u{ }}} \times {\text{CL}}_{{{\text{int}}}} }} $$

7 $$ {\text{CL}}_{{\text{h}}} ,{\text{mL}}/{\min}/{\text{kg}} = { }\frac{{{{Q}} \times {\text{CL}}_{{{\text{int}}}} { }}}{{{{Q}} + {\text{CL}}_{{{\text{int}}}} }} $$

8 $$ {\text{ER}}_{{\text{h}}} = { }\frac{{{\text{CL}}_{{\text{h}}} }}{{{Q }}} $$

*t*_1/2_ = in vitro half-life, *k* = slope of the linear regression fit, CL_int_ = intrinsic clearance, [V]incubation = incubation volume = 0.3 mL, [P]incubation = amount of S9 protein in the incubation = 0.6 mg, $\frac{{\left[ {{\text{Liver}}} \right]}}{{\left[ {{\text{BW}}} \right]}}$ = liver weight normalized by body weight = 26 g/kg (Davies and Morris 1993), SF = scaling factor S9 protein per gram of liver = 121 mg/g (Houston and Galetin 2008), CL_h_ = hepatic clearance, *Q* = hepatic blood flow rate in human = 20 mL/min/kg (Boxenbaum 1980), *f*_u_ = free fraction in plasma, and ER_h_ = hepatic extraction ratio.

PPB studies were done as described earlier (Fung et al. 2003; Mardal et al. 2016). Methanolic 4F-Cy-BAP and Fu-BAP solution (final concentration 0.5 µM) were spiked into fresh pooled human plasma (500 µL final volume). As human blood concentrations of both compounds were unknown, the selected plasma concentration was based on an average value of two intoxications with the synthetic opioid THF-F (Helander et al. 2017; Krotulski et al. 2018). After the incubation was conducted for 30 min at 37 °C, a volume of 100 µL (global approach, GA) was taken and transferred into a new reaction tube. The remaining sample was transferred into an ultrafiltrate device and centrifuged at 1600×*g* for 35 min. Thereafter, a volume of 100 µL of the ultrafiltrate (UF) was transferred to a new reaction tube. All samples were precipitated by adding a volume of 50 µL of ice-cold acetonitrile containing trimipramine-d_3_ (2.5 µM) as internal standard (IS). This was done as there was no deuterated 4F-Cy-BAP or Fu-BAP available and trimipramine-d_3_ was shown in be suitable as IS. Afterwards, they were cooled for 30 min at − 20 °C, centrifuged for 2 min at 18,407×*g*, and measured by LC-HRMS/MS. Ultrafiltration was done in triplicate.

Calculations of PPB were done using the following equations:

9 $$ f_{u} = \frac{{{\text{peak area ratio }}\left( {\frac{{4{\text{F}} - {\text{Cy}} - {\text{BAP}}_{{{\text{UF}}}} \,{\text{or}}\, {\text{Fu}} - {\text{BAP}}_{{{\text{UF}}}} }}{{{\text{IS}}_{{{\text{UF}}}} }}} \right)}}{{{\text{peak area ratio}} \left( {\frac{{4{\text{F}} - {\text{Cy}} - {\text{BAP}}_{{{\text{GA}}}} \,{\text{or}} \,{\text{Fu}} - {\text{BAP}}_{{{\text{GA}}}} }}{{{\text{IS}}_{{{\text{GA}}}} }}} \right)}} $$

10 $$ {\text{PPB}}, \% = \left( {1 - f_{u} } \right) \times 100 $$

### Isozyme mapping

As described elsewhere (Wagmann et al. 2016) with minor modifications, 4F-Cy-BAP and Fu-BAP (2.5 µM) were incubated with CYP1A2, CYP2A6, CYP2B6, CYP2C8, CYP2C9, CYP2C19, CYP2D6, CYP2E1, CYP3A4, CYP3A5 (50 pmol/mL each), FMO3 (0.25 mg protein/mL), respectively, or pHLM (1 mg protein/mL) as positive control for 30 min at 37 °C. All given concentrations are concentrations in the final incubation mixtures (100 µL final volume). In addition, the incubation mixtures contained 90 mM phosphate buffer (pH 7.4), 5 mM Mg^2+^, 5 mM isocitrate, 0.5 U/mL isocitrate dehydrogenase, 1.2 mM NADP^+^, and 200 U/mL superoxide dismutase. CYP2A6 and CYP2C9 incubations were conducted using Tris buffer instead of phosphate buffer, according to the manufacturer’s recommendation. In a preliminary test, reactions were started by adding the enzymes and stopped after 30 min by transferring a volume of 30 µL into new reactions tubes, which contained 10 µL ice-cold acetonitrile. Before analysis, the samples were centrifuged at 18,407×*g* for 5 min and the supernatants were transferred into autosampler vials. In a second test, only the involved isozymes and pHLM were incubated under identical conditions as described above (250 µL final volume). Reactions were stopped after 1, 5, 10, 15, 20, 25, and 30 min. Blank incubations without substrate and negative control incubations without enzymes were conducted to examine whether interfering or non-metabolically formed compounds were present. All incubations were done in duplicate.

### Maximum-tolerated concentration (MTC) studies in zebrafish larvae

Following the study of Richter et al. (2019a), zebrafish maintenance and all experiments with larvae were performed according to internal protocols based on standard methods (Westerfield 2007). Zebrafish larvae were raised at 28 °C in Danieau’s medium consisting of 17 mM NaCl, 2 mM KCl, 0.12 mM MgSO⁠_4_, 1.8 mM Ca(NO⁠_3_)⁠_2_, 1.5 mM HEPES, and 1.2 µM methylene blue. MTC studies were performed by placing the collected embryos in 6-well plates with 10 embryos per well in 2 mL Danieau’s medium. Zebrafish larvae at 4 days post-fertilization (dpf) were exposed to 4F-Cy-BAP and Fu-BAP dissolved in Danieau’s medium containing 1% (*v/v*) DMSO (waterborne exposition). Final compound concentrations were 0.01, 0.1, 1, 10, 50, and 100 µM. A negative control without drug was prepared, to exclude morphological malfunctions caused by DMSO (Xiong et al. 2017). The well plates remained over 24 h in the incubator at 28 °C. All drug exposure tests were done with 20 larvae. During exposure, the larvae were monitored using a LEICA M205 FA stereo microscope (Leica Mikrosysteme Vertrieb GmbH, Wetzlar, Germany).

### In vivo identification of metabolites

In compliance with an already described procedure (Richter et al. 2019a), 4F-Cy-BAP or Fu-BAP were administered to the zebrafish larvae (4 dpf) via medium. One well of a 6-well plate contained 10 zebrafish larvae and 2 mL of Danieau’s medium spiked with the compound (100 µM 4F-Cy-BAP or 80 µM Fu-BAP final concentrations, respectively). Drug exposure lasted for 24 h at 28 °C. Afterwards, the larvae and surrounding medium were collected separately and the medium was frozen at − 20 °C until use. Twenty larvae (from two wells) were transferred into a reaction tube, washed twice with 1 mL medium and euthanized by placing the tubes in ice water for 15 min. After the wash solution was removed, larvae were snap-frozen in liquid nitrogen, followed by lyophilization, and stored at − 20 °C until use.

Extraction of the medium was conducted by precipitation of 50 µL medium with 50 µL acetonitrile containing 0.1% (*v/v*) formic acid, shaking for 2 min, and cooling for 30 min at − 20 °C. Before analysis, the samples were centrifuged at 18,407×*g* for 2 min and the supernatant was transferred to an autosampler vial. Twenty larvae (one tube) were extracted with 50 µL methanol and shaken for 2 min. After centrifugation at 18,407×g for 2 min, the supernatant was transferred to an autosampler vial. All above described experiments were prepared and analyzed in triplicate. Blank zebrafish larvae (*n* = 2) were incubated in the medium without drugs and analyzed along with their blank medium to identify interfering compounds. Furthermore, a control medium sample containing only the drug in Danieau´s medium was prepared, respectively, for the detection of compound degradations during incubation.

### In vitro µ-opioid (MOR) receptor activity

To assess the in vitro biological activity of 4F-Cy-BAP and Fu-BAP, a live cell-based reporter assay was used that monitors functional complementation of a split nanoluciferase (NanoLuc Binary Technology) following agonist-induced recruitment of a β-arrestin 2 (βarr2) protein (fused to a small part of NanoLuc) to MOR (fused to a large part of NanoLuc). Details regarding the development of the stable cell line used here have been reported elsewhere (Cannaert et al. 2017, 2018).

Engineered HEK 293 T cells were routinely maintained at 37 °C, 5% CO_2_, under humidified atmosphere in DMEM supplemented with 10% heat-inactivated FBS, 100 U/mL of penicillin, 100 µg/mL of streptomycin and 0.25 µg/mL of amphotericin B. Stability of the cell lines was followed by flow cytometric analysis. For experiments, cells were plated on poly-D-lysine coated 96-well plates at 5 × 10^4^ cells/well and incubated overnight. The cells were washed twice with Opti-MEM® I Reduced serum medium to remove any remaining FBS and 100 µL of Opti-MEM® I was added. The Nano-Glo Live Cell reagent, a non-lytic detection reagent containing the cell permeable furimazine substrate, was prepared by diluting the Nano-Glo Live Cell substrate 20-fold using Nano-Glo LCS Dilution buffer, and 25 µL was added to each well. Subsequently, the plate was placed in the TriStar2 LB 942 multimode microplate reader (Berthold Technologies GmbH & Co., Germany). Luminescence was monitored during the equilibration period until the signal stabilized (15 min). We added 20 µL per well of test compounds, present as 6.75 × stocks (as 20 µL was added to 135 µL in total) in Opti-MEM® I. The luminescence was continuously measured for 120 min. Solvent controls were run in all experiments. Curve fitting and statistical analyses were performed using GraphPad Prism. The concentration–response curves were generated from experiments performed in triplicate, the data points representing the mean area under the curve (AUC) ± standard error of mean (SEM). All results were normalized to the maximal activity (*E*_max_) of hydromorphone (= 100%), used as the reference compound. Curve fitting of concentration − effect curves via nonlinear regression was employed to determine the potency (EC_50_) and the efficacy (*E*_max_).

### LC–HRMS/MS system

The used Thermo Fisher Scientific (TF, Dreieich, Germany) Dionex UltiMate 3000 RS pump was composed of a degasser, a quaternary pump, and an UltiMate autosampler and connected to a TF Q-Exactive Plus system equipped with a heated electrospray ionization source (HESI)-II. A volume of 1 µL was injected for all samples. Gradient elution was performed as described earlier (Helfer et al. 2015), using a TF Accucore PhenylHexyl column (100 mm × 2.1 mm, 2.6 μm). The composition of the mobile phases was: 2 mM aqueous ammonium formate containing formic acid (0.1%, *v/v*, pH 3, eluent A) and 2 mM ammonium formate solution with acetonitrile:methanol (1:1, *v/v*), water (1%, *v/v*), and formic acid (0.1%, *v/v*, eluent B). At first, the flow rate was set to 500 μL/min for a period of 10 min followed by 800 µL/min for 10–13.5 min. The gradient was stepped from 0 to 1 min hold 99% A, 1–10 min to 1% A, 10–11.5 min hold 1% A, and 11.5–13.5 min hold 99% A. HESI-II source settings were: heater temperature, 320 °C; ion transfer capillary temperature, 320 °C; spray voltage, 4.0 kV; ionization mode, positive; sheath gas, 60 arbitrary units (AU); auxiliary gas, 10 AU; sweep gas, 0 AU; and S-lens RF level, 50.0. External mass calibrations were done in advance before analysis as recommended by the manufacturer. Identification and quantification of parent compounds and metabolites were performed using full scan data and a subsequent data‐dependent MS^2^ (dd-MS^2^) mode with an inclusion list containing the exact masses of the respective parent compound and its presumed metabolites. Expected phase I metabolites such as hydroxy, dihydroxy or *N*-dealkyl metabolites and phase II e.g. sulfates, glucuronides were the inclusion lists. Full scan data acquisition was conducted as follows: resolution, 35,000; microscans, 1; automatic gain control (AGC) target, 1e^6^; maximum injection time (IT), 120 ms; and scan range, *m/z* 50–750. The following settings for the dd-MS^2^ mode were defined: option “pick others”, enabled; dynamic exclusion, disabled; resolution, 17,500; microscans, 1; isolation window, 1.0 mass-to-charge ratio (*m/z*); loop count, 5; AGC target, 2e^5^; maximum IT, 250 ms; high collision dissociation cell with stepped normalized collision energy, 17.5, 35.0, 52.5; exclude isotopes, on; spectrum data type, profile; and underfill ratio, 1%. Chemical structure drawings of presumed metabolites and exact mass calculations were prepared by ChemSketch 2010 12.01 (ACD/Labs, Toronto, Canada). Data handling was performed by TF Xcalibur Qual Browser software version 2.2. Automated peak integration settings were as follows: mass tolerance, 5 ppm; peak detection algorithm, INCOS; baseline window, 40; area noise factor, 5; and peak noise factor, 10.

## Results

### In vitro metabolic stability and PPB

Metabolic stability data are summarized in Table S1 in the Electronic Supplementary Material (ESM). Non-metabolic compound degradation during the pHLS9 incubations could be excluded by control incubations as the *t*-tests did not show a significant difference between the parent compound concentration after 360 min in control incubations and the initial concentrations after 1 min. Based on decreasing enzyme activities after 2 h of incubation, the cut-off value for determination of in vitro half-lives was defined to be 90 min (Baranczewski et al. 2006). Since the half-life of 4F-Cy-BAP was longer than 90 min, no clearance values and ER_h_ were calculated. The half-life of Fu-BAP was 71 min, resulting in a CL_int_ of 15 mL/min/kg. Calculations of *f*_u_ gave values of 0.02 for 4F-Cy-BAP and 0.05 in terms of Fu-BAP resulting in a PPB of 98% (4F-Cy-BAP) and 95% (Fu-BAP). CL_h_ predictions of Fu-BAP in consideration of *f*_u_ resulted in 0.7 mL/min/kg in both models. In disregard of *f*_u_, CL_h_ values were 10.6 mL/min/kg, calculated with the parallel tube and 8.6 mL/min/kg with the well-stirred model, which gave ER_h_ values of 0.5 and 0.4, respectively.

### Identification of in vitro and in vivo metabolites

**Fig. 2:**
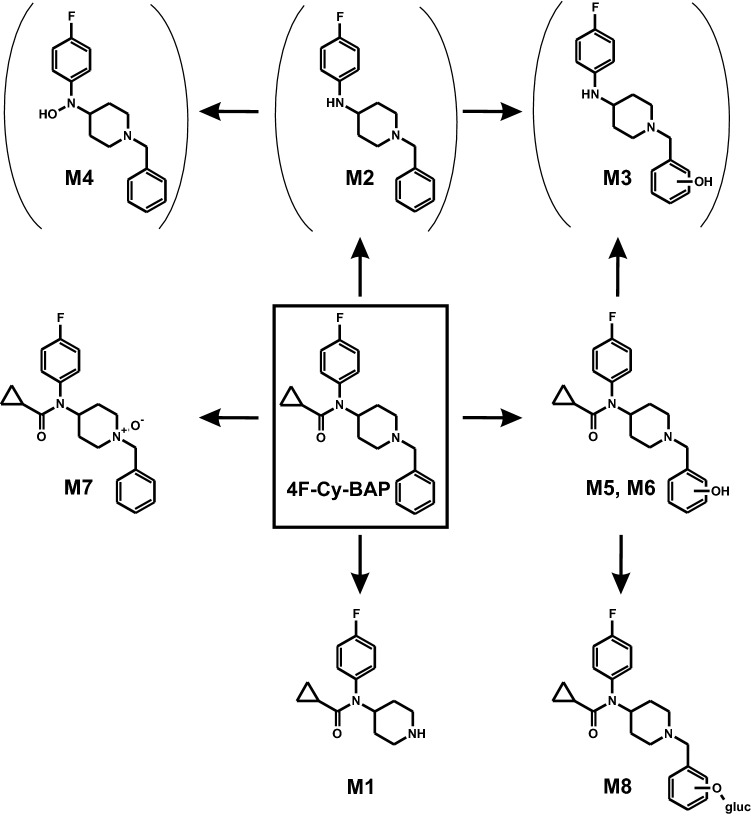
In vitro and in vivo metabolic pathways of 4F-Cy-BAP. Metabolites in brackets are considered to be artifacts

**Fig. 3:**
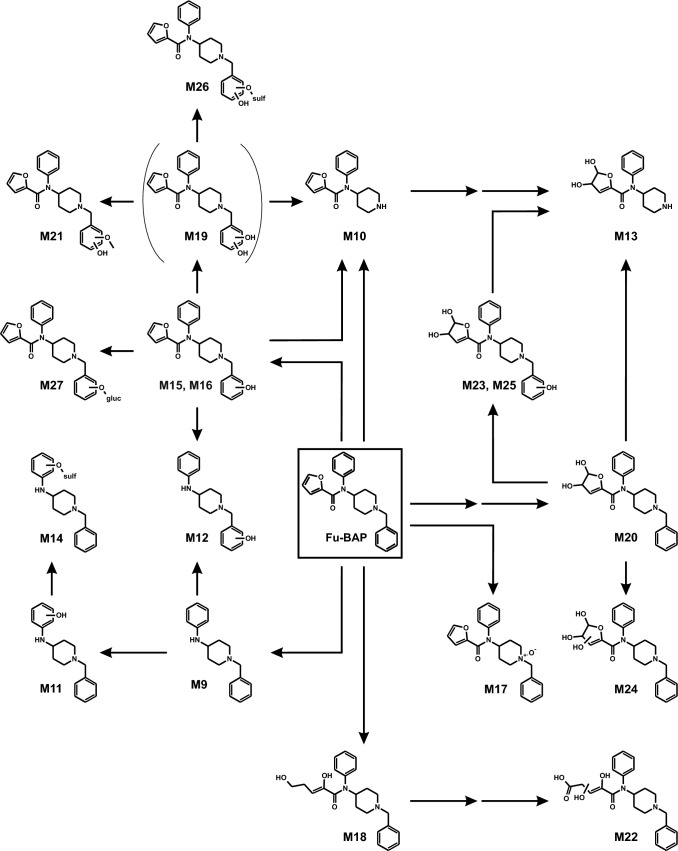
In vitro and in vivo metabolic pathways of Fu-BAP. For reasons of clarity, some arrows of expected metabolization steps are not shown. The metabolite in brackets is considered to be an artifact

**Fig. 4:**
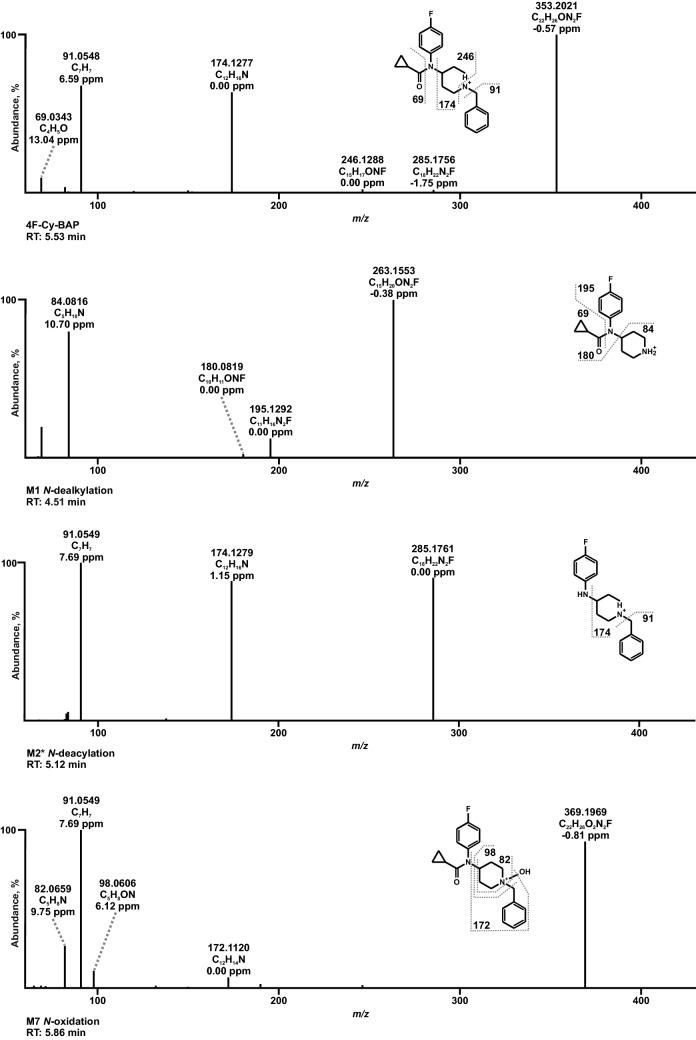
MS^2^ spectra of 4F-Cy-BAP and its most abundant metabolites identified in all investigated in vitro and in vivo models, sorted by increasing precursor ion masses and retention times (RT). The metabolite marked with an asterisk is considered to be an artifact

**Fig. 5:**
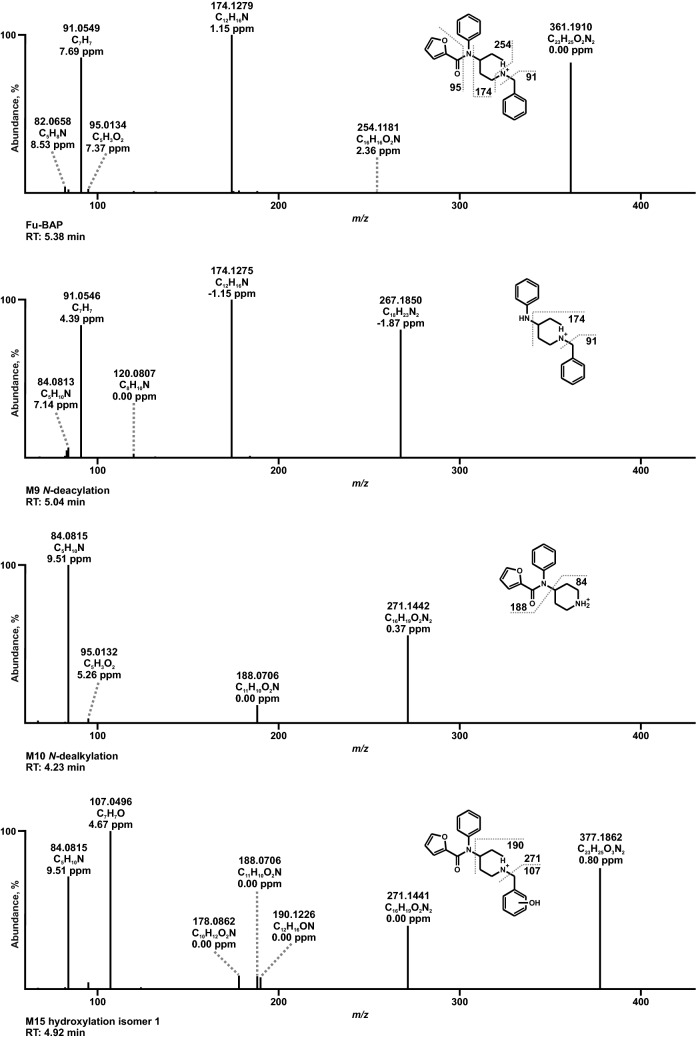
MS^2^ spectra of Fu-BAP and its most abundant metabolites identified in all investigated in vitro and in vivo models, sorted by increasing precursor ion masses and retention times (RT)

To avoid redundancies and to ease readability, results of in vitro and in vivo metabolite identification will be combined in the following section. Metabolites were identified by mining the data recorded in full-scan mode for their on beforehand calculated exact precursor ions (PIs). Subsequently, the spectra of the tentative metabolites obtained from the dd-MS^2^ mode were compared to that of the respective parent compound. All metabolites are listed in Table S2 in the ESM along with their metabolite ID, PI recorded in MS^1^, characteristic fragment ions (FIs) in MS^2^, relative intensities, calculated exact masses, elemental composition, mass deviation errors of the most abundant FIs, and retention time (RT). The in vitro and in vivo metabolic pathways of 4F-Cy-BAP and Fu-BAP are depicted in Fig. 2 and Fig. 3, respectively. In total, 7 phase I and 1 phase II metabolites of 4F-Cy-BAP and 15 phase I and 4 phase II metabolites in case of Fu-BAP were tentatively identified. The MS^2^ spectra of 4F-Cy-BAP or Fu-BAP and their three most abundant in vitro and in vivo metabolites are given in Figs. 4 and 5, respectively. In addition, the MS^2^ spectra of the lower abundant metabolites are represented in Fig. S1 (4F-Cy-BAP) and Fig. S2 (Fu-BAP) in the ESM.

In the following section, only exact masses will be used for the characterization of parent compounds and their respective metabolites. High abundant but less characteristic FIs of 4F-Cy-BAP (PI at *m/z* 353.2023) as well as Fu-BAP (PI at *m/z* 361.1910) were FIs at *m/z* 174.1277 and at *m/z* 91.0542. The former fragment originated from the benzyl piperidine part of the compounds and the latter of the phenyl coupled to the methyl spacer after piperidine cleavage. A distinctive fragment of 4F-Cy-BAP was the FI at *m/z* 246.1288, which was generated after the separation of the piperidine nitrogen plus benzyl part. Another prominent FI at *m/z* 69.0334 contained the cyclopropyl and carbonyl moiety formed after amide cleavage. Equally, distinguishing FIs of Fu-BAP were the less abundant FI at *m/z* 254.1175 and the FI at *m/z* 95.0127, which differed from the MS^2^ fragments of 4F-Cy-BAP through substitution of the cyclopropyl with the furanyl group.

One of the most abundant metabolites of 4F-Cy-BAP was M1 (PI at *m/z* 263.1554), which originated from *N*-dealkylation at the piperidine nitrogen. A characteristic FI was FI at *m/z* 180.0819, which consisted of the fluorophenyl linked to the cyclopropyl moiety. M2 (PI at *m/z* 285.1761), showed a similar fragmentation pattern as the parent compound, except for the missing FI at *m/z* 69.0334, which represented the cyclopropyl and carbonyl moiety. *N*-oxidation of the piperidine nitrogen led to the formation of M7 (PI at *m/z* 369.1972). The characteristic FI at *m/z* 98.0600 correlated with FI at *m/z* 84.0807 varying in one oxygen and two missing hydrogen atoms.

The Fu-BAP metabolite M9 (PI at *m/z* 267.1855) emerged from *N*-deacylation at the amide. Its MS^2^ spectrum was similar to that of the parent compound, except for the FI at *m/z* 95.0127, which originated from the furanyl part. M10 (PI at *m/z* 271.1441) was formed by *N*-dealkylation at the piperidine nitrogen and specified by FI at *m/z* 188.0706, which was generated after separation of the piperidine. M15 (PI at *m*/*z* 377.1859) was one of two hydroxy isomers, with the hydroxy group located at the phenyl part, which was part of the benzyl moiety. The FI at *m*/*z* 107.0491 corresponded to the FI at *m*/*z* 91.0548, which was altered by one oxygen atom. Both hydroxy isomers (M15, M16) were distinguishable from each other by different RT and intensities.

The *N*-deacyl hydroxy metabolite of 4F-Cy-BAP, M3 (PI at *m/z* 301.1710) was formed by *N*-deacylation at the amide and followed by hydroxylation at the phenyl, which was represented by the prominent FI at *m/z* 107.0491. *N*-Deacylation followed by *N*-oxidation of the linker nitrogen between piperidine and fluorophenyl led to the formation of M4 (PI at *m/z* 301.1710). A characteristic FI of M4 was FI at *m/z* 193.1135, which was matched with the fluorophenyl part linked to the piperidine ring with one double bond indicating loss of water. The two hydroxy isomers M5 and M6 (PI at *m/z* 369.1972) were formed by hydroxylation at the phenyl part. As already described for the Fu-BAP metabolite M15, M5 and M6 were characterized by the same FI at *m/z* 107.0491. A differentiation between both isomers was possible by different RT. The phase II metabolite M8 (PI at *m/z* 545.2293) was formed by glucuronidation of M5 or M6, which was also identified by the FI at *m/z* 107.0491.

M11 and M12 (PI at *m/z* 283.1804) were two Fu-BAP metabolites formed by *N*-deacylation plus hydroxylation. M12 was the equivalent *N*-deacyl metabolite of M15 or M16, which was characterized by FI at *m/z* 107.0491. The hydroxylation of M11 (PI at *m/z* 283.1804) occurred at the phenyl, which was designated by FI at *m/z* 192.1258 originating from elimination of the benzyl part. M13 (PI at *m/z* 305.1495) was formed by *N*-dealkylation and dihydrodiol formation by epoxidation of one double bond at the furanyl, followed by a non-enzymatic hydrolysis. Identification of M13 followed FI at *m/z* 166.0862, which contained the phenyl linked to a remaining part of the furanyl. M14 (PI at *m/z* 363.1373) stemmed from M11 through sulfation of the hydroxy group and their MS^2^ spectra were in accordance to each other. The second hydroxy isomer M16 (PI at *m/z* 377.1859) showed a similar fragmentation pattern as M15. M17 (PI at *m/z* 377.1859) originated from *N*-oxidation of the piperidine nitrogen, which was also specified with FI at *m/z* 98.0600, as already described for M7 of 4F-Cy-BAP. The 2,5-dihydroxypent-2-enal metabolite M18 (PI at *m/z* 381.2172) occurred through oxidative opening of the furan ring, which was identified by FI at *m/z* 363.2067 and FI at *m/z* 174.1277. The former fragment indicated a loss of water and due to the presence of the latter, which was unchanged compared to parent compound, the water loss was located at the opened furan ring. Aromatic dihydroxylation led to the formation of M19 (PI at *m/z* 393.1808), with the hydroxy groups located at the phenyl being part of the benzyl. The specific FI at *m/z* 123.0440 represented the dihydroxylated benzyl moiety, which differed in two oxygen atoms to the FI at *m/z* 91.0548. The dihydrodiol metabolite M20 (PI at *m/z* 395.1965) was characterized due to the absence of FI at *m/z* 95.0127, which was assigned to the furanyl part. M21 (PI at *m/z* 407.1965) occurred through methylation of one hydroxy group at the catechol structure of M19. The pronounced FI at *m/z* 137.0597 corresponded to FI at *m/z* 107.0491, which was altered by one additional methoxy group. The dihydroxy-5-oxopent-3-enoic acid metabolite M22 (PI at *m/z* 411.1914) originated from oxidative furan ring opening, oxidation of the terminal hydroxy group to carboxylic acid, and an additional hydroxylation at the opened side chain as described for the saturated furan ring of a fentanyl analogue (Kanamori et al. 2019). The low abundant FI at *m/z* 367.2016, which indicated an elimination of carbon dioxide, was used for its characterization. Based on its MS^2^ spectrum, the hydroxy group could be attached to position 2 or 3 at the chain, but the exact position was not locatable. M23, M24, and M25 (PI at *m/z* 411.1914) were three dihydrodiol-hydroxy isomers. In case of M23 and M25 the hydroxy group was determined at the phenyl, which belonged to the benzyl moiety, by means of FI at *m/z* 107.0491. Although the hydroxy group of M24 was located at the furanyl part, due to the presence of FI at *m/z* 267.1855, which resulted from the cleavage of the furanyl moiety, the precise structure of the furanyl residue was not determinable. Therefore, the most likely ring-closed structure is given for M24. The phase II metabolite M26 (PI at *m/z* 473.1376) was formed by sulfation of one hydroxy group of M21. It was specified by the FI at *m/z* 203.0008, which correlated to the FI at *m/z* 123.0440, differing in one sulfate group. M27 (PI at *m/z* 553.2180) was the corresponding phase II metabolite of M15 or M16, which was formed by glucuronidation of the hydroxy group. The prominent FI at *m/z* 107.0491 was used for identification. In the negative control incubations of all in vitro models and in the zebrafish larvae control media, the *N*-deacyl-metabolites M2 and M9 were also present with similar peak intensities as in the corresponding incubations with enzymes. However, in incubations without NADP^+^ solely the peak intensity of M9 increased. Blank incubations confirmed the absence of interfering compounds.

### Isozyme mapping

Blank incubations confirmed the absence of interfering compounds. The involvement of single isozymes compared to pHLS9 and pHLM incubations of both compounds is listed in Table S3 in the ESM.

*N*-Dealkyl 4F-Cy-BAP (M1) was present in incubations of CYP1A2, CYP2C19, CYP3A4, and CYP3A5. Furthermore, CYP2C19 catalyzed the formation of the two hydroxy isomers (M5, M6). The former (M5) was also formed by CYP2D6. CYP3A4 and CYP3A5 contributed to the emergence of the *N*-oxide (M7). Equally, several isozymes catalyzed the formation of the *N*-dealkyl Fu-BAP (M10), namely CYP1A2, CYP2C8, CYP2C19, CYP2D6, and CYP3A4. Apart from that, CYP2C8 was only involved in the formation of the *N*-oxide (M17). CYP2C19 contributed to numerous different steps, amongst them the formation of the two hydroxy isomers (M15, M16), the *N*-oxide (M17), and the furan ring opened 2,5-dihydroxypent-2-enal metabolite (M18). CYP2D6 was involved in the formation of the dihydrodiol metabolite (M20), the furan ring opened dihydroxy-5-oxopent-3-enoic acid metabolite (M22), as well as the dihydrodiol-hydroxy metabolite (M24). CYP3A4 was another considerable isozyme, catalyzing, besides M10 the formation of the hydroxy isomer (M15), the *N*-oxide (M17), and the dihydrodiol-hydroxy metabolite (M24).

### MTC studies in zebrafish larvae

**Fig. 6:**
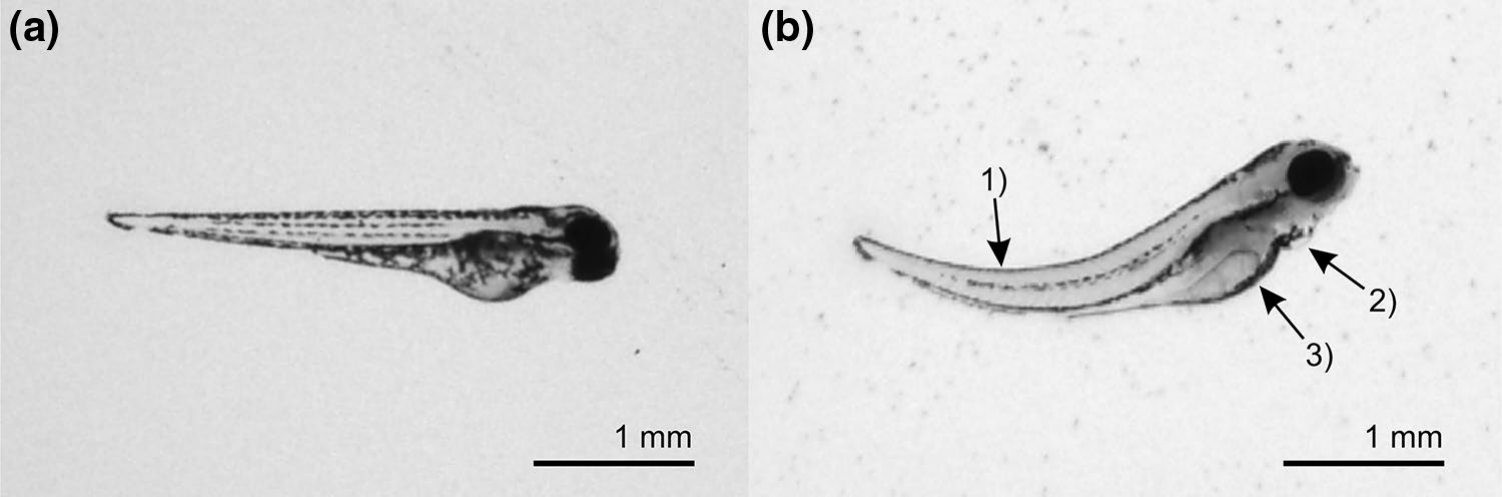
Microscopic image of two zebrafish larvae (**a**) in control medium (Danieau’s medium) plus 1% DMSO and (**b**) in Danieau´s medium containing 100 µM Fu-BAP plus 1% DMSO. Morphological malfunctions were a spinal curvature (1), abnormal pericardial edema (2), and a dark brown colored yolk sac (3)

Survival rates of the 4 dpf larvae exposed for 24 h to both compounds at concentrations of 0.01, 0.1, 1, 10, 50, and 100 µM were 100%. However, after treatment with 100 µM Fu-BAP, 80% of the larvae showed malformations and changes in behavior. An influence of DMSO could be excluded by the negative control incubation. Figure 6 shows a larva in control Danieau’s medium (a) or treated with 100 µM Fu-BAP (b), both containing 1% (*v/v*) DMSO. Visible morphological changes manifested in a spinal curvature (1), abnormal pericardial edema (2), and a dark brown colored yolk sac (3), which has already been described e.g. for environmental toxins (Seok et al. 2008). Furthermore, various larvae were observed, which showed random body vibrations, a fast heartbeat, and slow movements after touching. To prevent toxic effects in subsequent in vivo experiments, the Fu-BAP concentration was reduced to 80 µM for the metabolism study. However, still 20% of the larvae exhibited morphological/behavioral changes after exposure to 80 µM Fu-BAP in the metabolism study.

### Determination of the in vitro activity at MOR

**Fig. 7:**
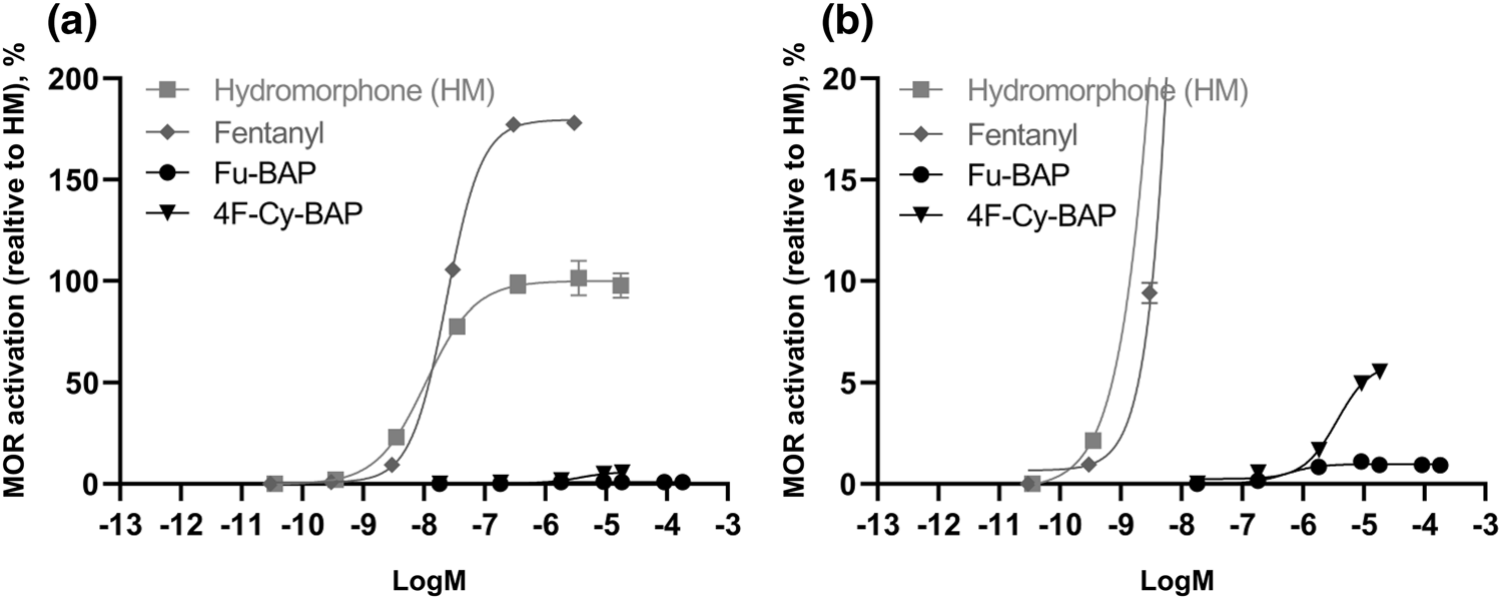
Concentration-dependent interaction of µ-opioid (MOR) receptor with β-arrestin 2 (βarr2) protein upon stimulation with hydromorphone (HM), fentanyl, 4F-Cy-BAP and Fu-BAP in (**a**) full concentration curves or (**b**) zoom on lower part of the graph. Data are given as mean receptor activation ± SEM (*n* = 3), normalized to the *E*_max_ (maximal activity) of HM (= 100%)

Analysis of the in vitro MOR activation potential of 4F-Cy-BAP and Fu-BAP showed an *E*_max_ of 5.98% and 0.98%, respectively, compared to the reference compound hydromorphone (*E*_max_ of 100%) and fentanyl (*E*_max_ of 180%), as presented in Fig. 7 and also summarized in Table S4 in the ESM along with their EC_50_ values.

## Discussion

### In vitro metabolic stability, predicted in vivo clearance, and PPB

Metabolic stability was characterized by *t*_1/2_, CL_int_, CL_h_, and ER_h_. CL_int_ differs from CL_h_ in the independence of physiological factors, such as hepatic blood flow and drug binding (Baranczewski et al. 2006). In vitro *t*_1/2_ was determined by decreasing 4F-Cy-BAP or Fu-BAP amounts during incubation with pHLS9 from 1 till 90 min. As several incubations were prepared at once, the first samples had to be taken at *t* = 1 min. CL_int_ of Fu-BAP was rated to be low in accordance to McNaney et al. (2008). No half-life and clearance values of 4F-Cy-BAP could be determined as it demonstrated only weak metabolic degradation.

CL_h_ was predicted by parallel tube and well-stirred model. The parallel tube model describes the liver as a set of tubes representing a sinusoid, with the drug concentration exponentially decreasing in the direction of the hepatic vein (Choi et al. 2019). However, the liver is considered as a single, well-mixed compartment with a fixed drug concentration in the well-stirred model (Segers et al. 2019). CL_h_ values of Fu-BAP were identical in both models by considering *f*_u_. CL_h_ predictions without *f*_u_ led to much higher values in both models.

ER_h_ estimations provide insight into the oral bioavailability of drugs under consideration of Q_h_ (Benet and Zia-Amirhosseini 1995). The calculated ER_h_ was based on CL_h_ values without *f*_u_ and it could be classified as intermediate in both models in accordance to Rogge and Taft (2009). As expected, no significant differences between both models were found, because this is rather the case for high ER_h_ drugs (Mehvar 2018).

Based on the free drug theory (Bohnert and Gan 2013), toxicokinetic effects of drugs, e.g. distribution or excretion, depends on their *f*_u_, which is strongly linked with their PPB. As both compounds showed a high PPB of more than 90%, a simultaneous intake with other drugs of abuse with similar high PPB such as cannabinoids (Mardal et al. 2016) or NBOMes (Richter et al. 2019b) could lead to an accumulation and adverse effects by displacement from the binding site. Moreover, a PPB higher than 70% is expected to have an impact on e.g. the clearance (Lindup and Orme 1981). However, another study indicated that in particular, clearance predictions of basic compounds based on in vitro measurement are more consistent with in vivo data regardless of any drug binding (Obach 1999). In addition, other influencing factors must be considered, e.g. active transport into the hepatocytes or elimination route (Smith et al. 2010).

### Comparison of in vitro and in vivo metabolites

Although in vitro metabolism studies may have some advantages, e.g. cost-effectivity or feasibility, there is often a discrepancy between the metabolites identified in vitro to those in human (Richter et al. 2019a). As no human samples after intake of 4F-Cy-BAP or Fu-BAP were available, an additional in vivo assay should confirm possible main targets for toxicological screenings. Comparison of the identified in vitro and in vivo metabolites in all investigated models of 4F-Cy-BAP and Fu-BAP are summarized in Table 1. The largest number of 4F-Cy-BAP and Fu-BAP metabolites (7 and 16, respectively) were identified in the zebrafish larvae extracts. These metabolites included all metabolites previously detected in the pHLS9 and pHLM incubations as well as in the zebrafish larvae media plus two novel 4F-Cy-BAP and eight Fu-BAP metabolites. However, all phase II metabolites were exclusively identified in zebrafish larvae extracts. This finding is due to the missing cofactors for the phase II enzymes in the pHLM incubations. In the case of pHLS9 incubations, the lower substrate concentration and shorter incubation time compared to the zebrafish larvae experiments are expected to be the main causes.

**Table 1:** List of 4F-Cy-BAP and Fu-BAP metabolites, respectively detected in zebrafish larvae incubations, pHLS9 or pHLM incubations, which were rated from + to +  +  + according to their absolute peak areas

Parent compound Metabolite ID	Metabolic reaction	In vivo		In vitro	
		Zebrafish larvae			
		Larvae extract 24 h	Medium 24 h	pHLS9 6 h	pHLM 0.5 h
4F-Cy-BAP					
M1	*N*-Dealkylation	** + + + **	** + **	** + + **	** + + **
M2*	*N*-Deacylation	** + + + **	** + + + **	** + **	** + **
M3*	*N*-Deacylation + hydroxylation	+ +	** + **	+	+
M4*	*N*-Deacylation + *N*-oxidation	** + + + **	N.D	N.D	N.D
M5	Hydroxylation isomer 1	+ +	+	N.D	N.D
M6	Hydroxylation isomer 2	N.D	N.D	N.D	N.D
M7	*N*-Oxidation	+ + +	N.D	** + **	** + + **
M8	Hydroxylation + glucuronidation	+	N.D	N.D	−
Fu-BAP					
M9	*N*-Deacylation	** + + + **	** + + **	** + + **	+ +
M10	*N*-Dealkylation	** + + + **	+	** + + **	** + + **
M11	*N*-Deacylation + hydroxylation isomer 1	+	N.D	** + + **	** + + **
M12	*N*-Deacylation + hydroxylation isomer 2	+ +	N.D	+	+
M13	*N-*Dealkylation + epoxidation + hydrolyze (dihydrodiol)	+	N.D	N.D	N.D
M14	*N*-Deacylation + hydroxylation + sulfation	+ +	N.D	N.D	−
M15	Hydroxylation isomer 1	** + + + **	** + **	+	+
M16	Hydroxylation isomer 2	N.D	N.D	N.D	N.D
M17	*N*-Oxidation	+ + +	N.D	+	** + + **
M18	Oxidation (furan ring open)	+ + +	** + **	N.D	+
M19*	Dihydroxylation	+	N.D	N.D	N.D
M20	Epoxidation + hydrolyze (dihydrodiol)	+ +	N.D	+ +	+
M21	Dihydroxylation + methylation	+ +	N.D	N.D	−
M22	Oxidation (furan ring open, carboxylic acid) + hydroxylation	N.D	N.D	N.D	N.D
M23	Epoxidation + hydrolyze + hydroxylation isomer 1	+	N.D	N.D	N.D
M24	Epoxidation + hydrolyze + hydroxylation isomer 2	N.D	N.D	N.D	N.D
M25	Epoxidation + hydrolyze + hydroxylation isomer 3	+	N.D	N.D	N.D
M26	Dihydroxylation + sulfation	+	N.D	N.D	−
M27	Hydroxylation + glucuronidation	+ +	N.D	N.D	−

Metabolite IDs correspond to Table S2 in the ESM

*N.D.* not detected

*Considered to be artifacts, — formation with the given incubation conditions not possible

**Fig. 8:**
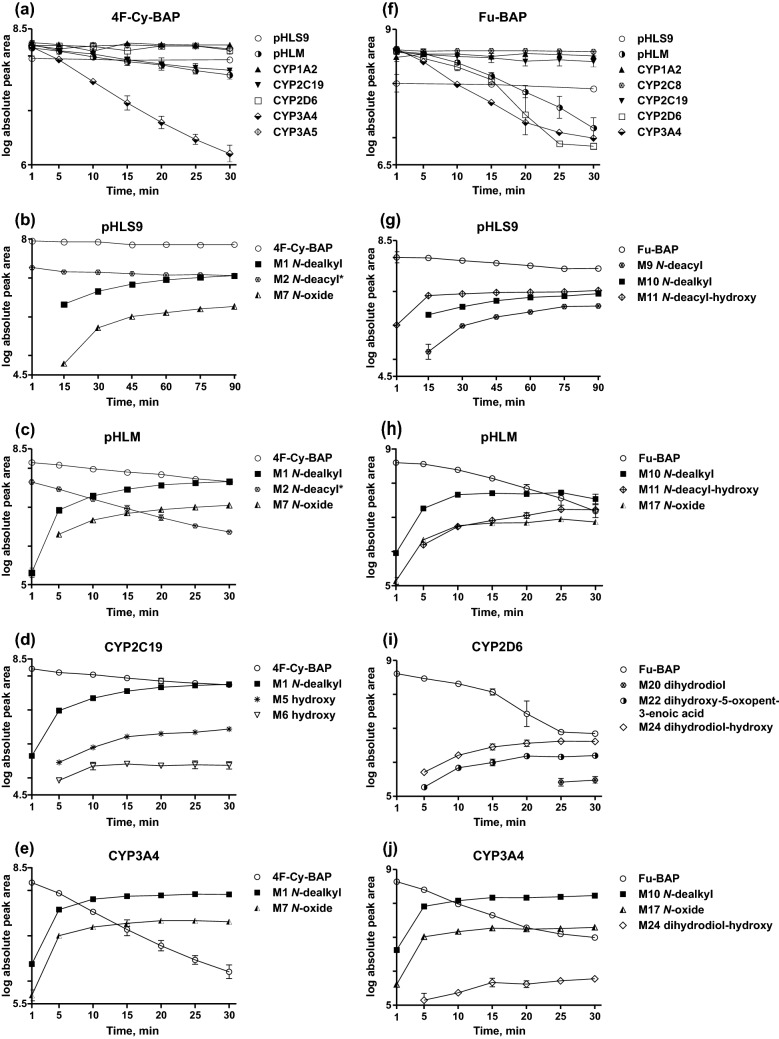
Changes of the amount of parent compounds in pHLS9, pHLM, and isozyme incubations (**a**) 4F-Cy-BAP and (**f**) Fu-BAP are presented as changes in the logarithm of the absolute peak areas as a function of time (min). The formation rates of the three most abundant metabolites are depicted in comparison to the changes of the logarithmic absolute peak areas of parent compounds (4F-Cy-BAP, **b**–**e**) and (Fu-BAP, **g**–**j**) in pHLS9 **b**, **g**, pHLM **c**, **h**, CYP2C19 **d**, CYP2D6 **i** or CYP3A4 **e**, **j**. The metabolite marked with an asterisk is considered to be an artifact. If data points are missing, no signal was detected

As shown in Fig. 8, the in vitro formation of the *N*-deacyl-metabolite of 4F-Cy-BAP (M2) had its peak already within the first minute of incubation and declined afterwards most likely due to further biotransformation to M3. However, its formation was also observed in stock solutions after long-term storage and negative control incubations and thus, it may be also of artificial nature. Its subsequent metabolites (M3, M4) were also marked to be possible artifacts e.g. in Table 1. Similar findings were observed for Fu-BAP. However, based on an increasing peak intensity of M9 in absence of NADP^+^, M9 and all metabolites derived thereof were presented as metabolites. Some metabolites (M6, M16, M22, M24) were solely detected in single isozyme incubations and therefore considered as minor metabolites. Moreover, it can be assumed that the origin of the Fu-BAP metabolite M19 was artificial because the retention time correlated with that of M26. Nevertheless, M19 was obliged to be a precursor of the phase II metabolites M21 and M26, but most probably with another RT. Suitable analytical targets for toxicological urine screenings should be the *N*-dealkyl metabolites (M1, M10) and the *N*-deacyl metabolites (M2, M9) of both compounds, and additionally 4F-Cy-BAP *N*-oxide (M7), as well as hydroxy Fu-BAP (M15).

### Involvement of CYP2D6, CYP3A4, and other isozymes in phase I steps

Isozyme mapping is essential for the prediction of possible interactions, e.g. drug-drug interactions, or interindividual variations by different expressions of isozymes. Figure 8 summarizes the change in the amount of each parent compound in the incubations of pHLS9, pHLM, and in incubations of all involved isozymes. The formation rates of the three most abundant metabolites and the change in amount of each parent compound in pHLS9, pHLM, CYP2C19 or CYP2D6, and CYP3A4 incubations are also given in Fig. 8. In particular, 4F-Cy-BAP was mainly metabolized by CYP3A4, which may result in increased drug levels and intoxications after co-consumption of CYP3A4 inhibitors, e.g. tryptamines (Dinger et al. 2016). Due to the additional involvement of CYP2D6 in the Fu-BAP metabolism, inhibition of CYP3A4 is expected to be less substantial if the user is not a CYP2D6 poor metabolizer.

### In vitro MOR receptor activity

The receptor activation was evaluated via the interaction between βarr2, a cytosolic protein, and the G-protein coupled MOR. Both βarr2 and MOR are fused to an inactive part of nanoluciferase. When MOR is activated by a ligand, βarr2 is recruited to the receptor, allowing interaction of the complementary nanoluciferase subunits, yielding a functional enzyme that generates a bioluminescent signal in the presence of the substrate furimazine (Cannaert et al. 2019). In vitro MOR activity analysis of 4F-Cy-BAP and Fu-BAP revealed that these compounds were only able to activate MOR to a limited extent. Also the EC_50_ values of both compounds were strongly reduced compared to hydromorphone and fentanyl.

These findings are not surprising as in vivo studies in mice and rat showed that the replacement of the *N*-phenethyl group with a *N*-benzyl group resulted in a strong reduction in anti-nociceptive activity (Casy et al. 1969; Casy and Huckstep 1988). Moreover, the *N*-benzyl analog of fentanyl (benzylfentanyl) was originally listed in the US as a Scheduled I controlled substance, but was removed from the list as the Drug Enforcement Administration (DEA) indicated that this compound was inactive at MOR (DEA and DoJ 2010), in line with our unpublished findings.

## Conclusion

The current study focused on the toxicokinetic and toxicodynamic properties of the fentanyl homologs 4F-Cy-BAP and Fu-BAP. As 4F-Cy-BAP was metabolically much more stable with an in vitro t_1/2_ greater than 90 min, no clearances and ER_h_ were calculated. Predicted CL_int_ and ER_h_ values of Fu-BAP were classified as low and intermediate, respectively. The higher in vitro metabolic stability of 4F-Cy-BAP was confirmed by a smaller number of metabolites formed in vitro and in vivo in comparison to Fu-BAP. Overall, seven phase I and one phase II metabolites of 4F-Cy-BAP and 15 phase I and four phase II metabolites for Fu-BAP were identified, with the majority detected in zebrafish larvae. In particular, *N*-dealkylation, hydroxylation, *N*-oxidation, and *N*-deacylation were the main metabolic reactions. Therefore, these metabolites should be considered as useful targets for toxicological urine screenings. CYP3A4 and, in the case of Fu-BAP, additionally CYP2D6, were the two isozymes mainly involved in their in vitro phase I metabolism. Based on these findings, drug-drug interactions leading to CYP3A4 inhibition may cause an accumulation especially of 4F-Cy-BAP. CYP2D6 poor metabolizers could be equally affected by drug-drug interactions after intake of Fu-BAP. A simultaneous intake together with high protein bound drugs could lead to adverse reactions by displacement from the binding site. Treatment of larvae with Fu-BAP revealed malformations and changes in behavior. Only a weak activity at MOR (*E*_max_ values of 5.98% and 0.98% compared to HM, respectively) could be observed in vitro but strong agonism or antagonism at other receptors such as the sigma1, sigma2 or acetylcholine M2 and M3 receptors cannot be excluded and should be investigated.

## Electronic supplementary material

Below is the link to the electronic supplementary material.

Supplementary file1 (DOCX 1680 kb)
