# Supplementary material for: Toxicokinetics and toxicodynamics of the fentanyl homologs cyclopropanoyl-1-benzyl-4´-fluoro-4-anilinopiperidine and furanoyl-1-benzyl-4-anilinopiperidine
Source: Arch Toxicol. 2020 Apr 5;94(6):2009–25. doi: 10.1007/s00204-020-02726-1 (PMC7303074; doi:10.1007/s00204-020-02726-1)

**Archives of Toxicology**

**Electronic Supplementary Material**

**Toxicokinetics and toxicodynamics of the fentanyl homologs cyclopropanoyl-1-benzyl-4´-fluoro-4-anilinopiperidine and furanoyl-1-benzyl-4-anilinopiperidine**

**Tanja M. Gampfer, Lea Wagmann, Yu Mi Park, Annelies Cannaert, Jennifer Herrmann, Svenja Fischmann, Folker Westphal, Rolf Müller, Christophe P. Stove, Markus R. Meyer**

**Table S1** In vitro metabolic stability data of 4F-Cy-BAP and Fu-BAP including free fraction in plasma (*f_u_*), plasma protein binding (PPB), half-life (*t*_1/2_), in vitro intrinsic clearance (CL_int_), predicted in vivo clearance values using the well-stirred and parallel tube models (CL_h_) with and without considering *f*_u_, and hepatic extraction ratios (ER_h_).

| Compound | *f*_u_ | PPB [%] | *t*_1/2_ [min] | CL_int_  [mL/min/kg] | CL_h_  [mL/min/kg] | CL_h_  without *f*_u_ [mL/min/kg] | ER_h_  without *f*_u_ | CL_h_  [mL/min/kg] | CL_h_  without *f*_u_  [mL/min/kg] | ER_h_  without *f*_u_ |
| --- | --- | --- | --- | --- | --- | --- | --- | --- | --- | --- |
|  |  |  |  |  | well-stirred | | | parallel tube | | |
| 4F-Cy-BAP | 0.02 | 98 | > 90 | - | - | - | - | - | - | - |
| Fu-BAP | 0.05 | 95 | 71 | 15 | 0.7 | 8.6 | 0.4 | 0.7 | 10.6 | 0.5 |

**Table S2** List of 4F-Cy-BAP, Fu-BAP and all their metabolites with precursor ion (PI) masses recorded in MS^1^, characteristic fragment ions (FIs) in MS^2^, relative intensities in MS^2^, calculated exact masses, elemental composition, deviation from measured to calculated masses, and retention time (RT), respectively. The metabolites were sorted by increasing mass and RT. Metabolites marked with an asterisk are considered to be artifacts.

| *Parent compound*  Metabolite ID | Metabolic reaction | Measured masses PI and characteristic FIs [*m/z*] | Relative intensity in MS^2^ [%] | Calculated exact masses, [*m/z*] | Elemental composition | Mass deviation [ppm] | RT [min] |
| --- | --- | --- | --- | --- | --- | --- | --- |
| *4F-Cy-BAP* |  | PI at *m/z* 353.2021  FI at *m/z* 285.1756  FI at *m/z* 246.1288  FI at *m/z* 174.1277  FI at *m/z* 150.0713  FI at *m/z* 91.0548  FI at *m/z* 82.0658  FI at *m/z* 69.0651 | 100  2  2  63  2  67  4  10 | 353.2023  285.1761  246.1288  174.1277  150.0713  91.0542  82.0651  69.0334 | C_22_H_26_ON_2_F  C_18_H_22_N_2_F  C_15_H_17_ONF  C_12_H_16_N  C_9_H_9_NF  C_7_H_7_  C_5_H_8_N  C_4_H_5_O | -0.57  -1.75  0.00  0.00  0.00  6.59  8.53  13.04 | 5.53 |
| M1 | *N*-Dealkylation | PI at *m/z* 263.1553 FI at *m/z* 195.1292  FI at *m/z* 180.0819  FI at *m/z* 84.0816  FI at *m/z* 69.0344 | 100  12  3  79  19 | 263.1554  195.1292  180.0819  84.0807  69.0334 | C_15_H_19_ON_2_F  C_11_H_16_N_2_F  C_10_H_11_ONF  C_5_H_10_N  C_4_H_5_O | -0.38  0.00  0.00  10.70  14.49 | 4.51 |
| M2* | *N*-Deacylation | PI at *m/z* 285.1761  FI at *m/z* 174.1279  FI at *m/z* 91.0549  FI at *m/z* 84.0815 | 88  89  100  6 | 285.1761  174.1277  91.0542  84.0807 | C_18_H_22_N_2_F  C_12_H_16_N  C_7_H_7_  C_5_H_10_N | 0.00  1.15  7.69  9.51 | 5.12 |
| M3* | *N*-Deacylation + hydroxylation | PI at *m/z* 301.1710  FI at *m/z* 195.1292  FI at *m/z* 190.1226  FI at *m/z* 107.0496  FI at *m/z* 84.0815 | 44  17  11  100  37 | 301.1710  195.1292  190.1226  107.0491  84.0807 | C_18_H_22_ON_2_F  C_11_H_16_N_2_F  C_12_H_16_ON  C_7_H_7_O  C_5_H_10_N | 0.00  0.00  0.00  4.67  9.51 | 4.49 |
| M4* | *N*-Deacylation + *N*-oxidation | PI at *m/z* 301.1711  FI at *m/z* 193.1136  FI at *m/z* 150.0714  FI at *m/z* 132.0808  FI at *m/z* 100.0762  FI at *m/z* 91.0549  FI at *m/z* 82.0658 | 72  4  6  2  1  100  4 | 301.1710  193.1135  150.0713  132.0807  100.0756  91.0542  82.0651 | C_18_H_22_ON_2_F  C_11_H_14_N_2_F  C_9_H_9_NF  C_9_H_10_N  C_5_H_10_ON  C_7_H_7_  C_5_H_8_N | 0.33  0.52  0.67  0.76  6.00  7.69  8.53 | 5.63 |
| M5 | Hydroxylation isomer 1 | PI at *m/z* 369.1969  FI at *m/z* 263.1551  FI at *m/z* 195.1293  FI at *m/z* 190.1231  FI at *m/z* 107.0495  FI at *m/z* 84.0814  FI at *m/z* 69.0343 | 85  48  5  4  100  45  5 | 369.1972  263.1554  195.1292  190.1226  107.0491  84.0807  69.0334 | C_22_H_26_O_2_N_2_F  C_15_H_20_ON_2_F  C_11_H_16_N_2_F  C_12_H_16_N_2_F  C_7_H_7_O  C_5_H_10_N  C_4_H_5_O | -0.81  -1.14  0.51  2.63  3.74  8.33  13.04 | 5.15 |
| M6 | Hydroxylation isomer 2 | PI at *m/z* 369.1974  FI at *m/z* 263.1554  FI at *m/z* 195.1300  FI at *m/z* 190.1228  FI at *m/z* 152.1071  FI at *m/z* 107.0496  FI at *m/z* 84.0815  FI at *m/z* 69.0345 | 100  64  8  11  5  55  72  9 | 369.1972  263.1554  195.1292  190.1226  152.1069  107.0491  84.0807  69.0334 | C_22_H_26_O_2_N_2_F  C_15_H_20_ON_2_F  C_11_H_16_N_2_F  C_12_H_16_N_2_F  C_9_H_14_ON  C_7_H_7_O  C_5_H_10_N  C_4_H_5_O | 0.54  0.00  4.10  1.05  1.31  4.67  9.51  15.93 | 5.55 |
| M7 | *N*-Oxidation | PI at *m/z* 369.1969  FI at *m/z* 246.1290  FI at *m/z* 190.1225  FI at *m/z* 172.1120  FI at *m/z* 98.0606  FI at *m/z* 91.0549  FI at *m/z* 82.0659  FI at *m/z* 69.0343 | 93  2  3  7  11  100  27  2 | 369.1972  246.1288  190.1226  172.1120  98.0600  91.0542  82.0651  69.0334 | C_22_H_26_O_2_N_2_F  C_15_H_17_ONF  C_12_H_16_ON  C_12_H_14_N  C_5_H_8_ON  C_7_H_7_  C_5_H_8_N  C_4_H_5_O | -0.81  0.81  -0.53  0.00  6.12  7.69  9.75  13.04 | 5.86 |
| M8 | Hydroxylation + glucuronidation | PI at *m/z* 545.2293  FI at *m/z* 369.1973  FI at *m/z* 263.1553  FI at *m/z* 195.1300  FI at *m/z* 107.0496  FI at *m/z* 84.0815 | 37  2  13  1  100  7 | 545.2293  369.1972  263.1554  195.1292  107.0491  84.0807 | C_28_H_34_O_8_N_2_F  C_22_H_26_O_2_N_2_F  C_15_H_20_ON_2_F  C_11_H_16_N_2_F  C_7_H_7_O  C_5_H_10_N | 0.00  0.27  -0.38  4.10  4.67  9.51 | 4.58 |
| *Fu-BAP* |  | PI at *m/z* 361.1910  FI at *m/z* 254.1181  FI at *m/z* 178.0860  FI at *m/z* 174.1279  FI at *m/z* 95.0134  FI at *m/z* 91.0549  FI at *m/z* 84.0815  FI at *m/z* 82.0658 | 82  1  2  100  3  86  2  4 | 361.1910  254.1175  178.0862  174.1277  95.0127  91.0542  84.0807  82.0651 | C_23_H_25_O_2_N_2_  C_16_H_16_O_2_N  C_10_H_12_O_2_N  C_12_H_16_N  C_5_H_3_O_2_  C_7_H_7_  C_5_H_10_N  C_5_H_8_N | 0.00  2.36  -1.12  1.15  7.37  7.69  9.51  8.53 | 5.38 |
| M9 | *N*-Deacylation | PI at *m/z* 267.1850  FI at *m/z* 174.1275  FI at *m/z* 120.0807  FI at *m/z* 91.0546  FI at *m/z* 84.0813  FI at *m/z* 83.0735 | 80  100  3  82  6  5 | 267.1855  174.1277  120.0807  91.0542  84.0807  83.0729 | C_18_H_23_N_2_  C_12_H_16_N  C_8_H_10_N  C_7_H_7_  C_5_H_10_N  C_5_H_9_N | -1.87  -1.15  0.00  4.39  7.14  7.22 | 5.04 |
| M10 | *N*-Dealkylation | PI at *m/z* 271.1442  FI at *m/z* 188.0706  FI at *m/z* 95.0132  FI at *m/z* 84.0815 | 55  12  3  100 | 271.1441  188.0706  95.0127  84.0807 | C_16_H_18_O_2_N_2_  C_11_H_10_O_2_N  C_5_H_3_O  C_5_H_10_N | 0.37  0.00  5.26  9.51 | 4.23 |
| M11 | *N*-Deacylation + hydroxylation isomer 1 | PI at *m/z* 283.1808  FI at *m/z* 192.1258  FI at *m/z* 174.1280  FI at *m/z* 91.0549  FI at *m/z* 84.0816  FI at *m/z* 83.0738 | 90  7  100  74  3  11 | 283.1804  192.1257  174.1277  91.0542  84.0807  83.0729 | C_18_H_23_ON_2_  C_11_H_16_ON_2_  C_12_H_16_N  C_7_H_7_  C_5_H_10_N  C_5_H_9_N | 1.41  0.52  1.72  7.69  10.70  10.83 | 2.99 |
| M12 | *N*-Deacylation + hydroxylation isomer 2 | PI at *m/z* 283.1801  FI at *m/z* 190.1226  FI at *m/z* 177.1386  FI at *m/z* 107.0496  FI at *m/z* 84.0815 | 40  16  16  100  43 | 283.1804  190.1226  177.1386  107.0491  84.0807 | C_18_H_23_ON_2_  C_12_H_16_ON  C_11_H_17_N_2_  C_7_H_7_O  C_5_H_10_N | -1.06  0.00  0.00  4.67  9.51 | 4.36 |
| M13 | *N-*Dealkylation + epoxidation + hydrolyze | PI at *m/z* 305.1499  FI at *m/z* 287.1383  FI at *m/z* 194.0812  FI at *m/z* 166.0864  FI at *m/z* 84.0815 | 45  2  3  5  100 | 305.1495  287.1390  194.0811  166.0862  84.0807 | C_16_H_21_O_4_N_2_  C_16_H_19_O_3_N_2_  C_10_H_12_O_3_N  C_9_H_12_O_2_N  C_5_H_10_N | 1.31  -2.44  0.52  1.20  9.51 | 3.25 |
| M14 | *N*-Deacylation + hydroxylation + sulfation | PI at *m/z* 363.1366  FI at *m/z* 283.1800  FI at *m/z* 192.1256  FI at *m/z* 174.1278  FI at *m/z* 91.0548  FI at *m/z* 84.0814  FI at *m/z* 83.0736 | 69  37  5  100  95  4  9 | 363.1373  283.1804  192.1257  174.1277  91.0542  84.0807  83.0729 | C_18_H_23_O_4_N_2_S  C_18_H_23_ON_2_  C_11_H_16_ON_2_  C_12_H_16_N  C_7_H_7_  C_5_H_10_N  C_5_H_9_N | -1.93  -1.41  -0.52  0.57  6.59  8.33  8.43 | 3.68 |
| M15 | Hydroxylation isomer 1 | PI at *m/z* 377.1862  FI at *m/z* 271.1441  FI at *m/z* 190.1226  FI at *m/z* 188.0706  FI at *m/z* 178.0862  FI at *m/z* 107.0496  FI at *m/z* 95.0132  FI at *m/z* 84.0815 | 76  40  8  9  9  100  4  72 | 377.1859  271.1441  190.1226  188.0706  178.0862  107.0491  95.0127  84.0807 | C_23_H_25_O_3_N_2_  C_16_H_19_O_2_N_2_  C_12_H_16_ON  C_11_H_10_O_2_N  C_10_H_12_O_2_N  C_7_H_7_O  C_5_H_3_O_2_  C_5_H_10_N | 0.80  0.00  0.00  0.00  0.00  4.67  5.26  9.51 | 4.92 |
| M16 | Hydroxylation isomer 2 | PI at *m/z* 377.1859  FI at *m/z* 271.1439  FI at *m/z* 190.1225  FI at *m/z* 188.0705  FI at *m/z* 178.0860  FI at *m/z* 107.0494  FI at *m/z* 95.0131  FI at *m/z* 84.0814 | 76  17  59  4  5  100  3  33 | 377.1859  271.1441  190.1226  188.0706  178.0862  107.0491  95.0127  84.0807 | C_23_H_25_O_3_N_2_  C_16_H_19_O_2_N_2_  C_12_H_16_ON  C_11_H_10_O_2_N  C_10_H_12_O_2_N  C_7_H_7_O  C_5_H_3_O_2_  C_5_H_10_N | 0.00  -0.74  -0.53  -0.53  -1.12  2.80  4.21  8.33 | 5.10 |
| M17 | *N*-Oxidation | PI at *m/z* 377.1862  FI at *m/z* 278.1176  FI at *m/z* 219.1127  FI at *m/z* 190.1225  FI at *m/z* 188.0706  FI at *m/z* 172.1121  FI at *m/z* 146.0965  FI at *m/z* 100.0762  FI at *m/z* 98.0605  FI at *m/z* 95.0132  FI at *m/z* 91.0548  FI at *m/z* 82.0658 | 75  3  2  5  2  6  4  4  11  2  100  26 | 377.1859  278.1175  219.1128  190.1226  188.0706  172.1120  146.0964  100.0756  98.0600  95.0127  91.0542  82.0651 | C_23_H_25_O_3_N_2_  C_18_H_16_O_2_N  C_12_H_15_O_2_N_2_  C_12_H_16_ON  C_11_H_10_O_2_N  C_12_H_14_N  C_10_H_12_N  C_5_H_10_ON  C_5_H_8_ON  C_5_H_3_O_2_  C_7_H_7_  C_5_H_8_N | 0.80  0.36  -0.46  -0.53  0.00  0.58  0.68  6.00  5.10  5.26  6.59  8.53 | 5.73 |
| M18 | Oxidation (furan ring open) | PI at *m/z* 381.2172  FI at *m/z* 363.2068  FI at *m/z* 180.1019  FI at *m/z* 174.1278  FI at *m/z* 172.1121  FI at *m/z* 91.0548  FI at *m/z* 84.0814  FI at *m/z* 82.0658 | 84  5  2  79  8  100  3  4 | 381.2172  363.2067  180.1019  174.1277  172.1120  91.0542  84.0807  82.0651 | C_23_H_29_O_3_N_2_  C_23_H_27_O_2_N_2_  C_10_H_14_O_2_N  C_12_H_16_N  C_12_H_14_N  C_7_H_7_  C_5_H_10_N  C_5_H_8_N | 0.00  0.28  0.00  0.57  0.58  6.59  8.33  8.53 | 4.63 |
| M19* | Dihydroxylation | PI at *m/z* 393.1807  FI at *m/z* 271.1440  FI at *m/z* 206.1179  FI at *m/z* 188.0707  FI at *m/z* 178.0863  FI at *m/z* 123.0443  FI at *m/z* 95.0131  FI at *m/z* 84.0815 | 81  58  5  13  8  100  6  99 | 393.1808  271.1441  206.1175  188.0706  178.0862  123.0440  95.0127  84.0807 | C_23_H_25_O_4_N_2_  C_16_H_19_O_2_N_2_  C_12_H_16_O_2_N  C_11_H_10_O_2_N  C_10_H_12_O_2_N  C_7_H_7_O_2_  C_5_H_3_O_2_  C_5_H_10_N | -0.25  -0.37  1.94  0.53  0.56  2.44  4.21  9.51 | 5.10 |
| M20 | Epoxidation + hydrolyze (dihydrodiol) | PI at *m/z* 395.1966  FI at *m/z* 174.1278  FI at *m/z* 166.0863  FI at *m/z* 132.0808  FI at *m/z* 91.0548  FI at *m/z* 84.0814  FI at *m/z* 82.0658 | 78  74  1  2  100  4  3 | 395.1965  174.1277  166.0862  132.0807  91.0542  84.0807  82.0651 | C_23_H_27_O_4_N_2_  C_12_H_16_N  C_9_H_12_O_2_N  C_9_H_10_N  C_7_H_7_  C_5_H_10_N  C_5_H_8_N | 0.25  0.57  0.60  0.76  6.59  8.33  8.53 | 4.70 |
| M21 | Dihydroxylation + methylation | PI at *m/z* 407.1966  FI at *m/z* 271.1441  FI at *m/z* 188.0706  FI at *m/z* 178.0861  FI at *m/z* 137.0598  FI at *m/z* 122.0364  FI at *m/z* 84.0814 | 34  39  6  2  100  3  39 | 407.1965  271.1441  188.0706  178.0862  137.0597  122.0362  84.0807 | C_24_H_27_O_4_N_2_  C_16_H_19_O_2_N_2_  C_11_H_10_O_2_N  C_10_H_12_O_2_N  C_8_H_9_O_2_  C_7_H_6_O_2_  C_5_H_10_N | 0.25  0.00  0.00  -0.56  0.73  1.64  8.33 | 5.06 |
| M22 | Oxidation (furan ring open, carboxylic acid) + hydroxylation | PI at *m/z* 411.1910  FI at *m/z* 367.2011  FI at *m/z* 323.1749  FI at *m/z* 174.1276  FI at *m/z* 132.0809  FI at *m/z* 91.0549  FI at *m/z* 84.0815  FI at *m/z* 82.0658 | 77  1  5  78  2  100  3  4 | 411.1914  367.2016  323.1754  174.1277  132.0807  91.0542  84.0807  82.0651 | C_23_H_27_O_5_N_2_  C_22_H_27_O_3_N_2_  C_20_H_23_O_2_N_2_  C_12_H_16_N  C_9_H_10_N  C_7_H_7_  C_5_H_10_N  C_5_H_8_N | -0.97  -1.36  -1.55  -0.57  1.51  7.69  9.51  8.53 | 4.02 |
| M23 | Epoxidation + hydrolyze + hydroxylation isomer 1 | PI at *m/z* 411.1912  FI at *m/z* 305.1494  FI at *m/z* 194.0817  FI at *m/z* 190.1232  FI at *m/z* 174.1281  FI at *m/z* 166.0863  FI at *m/z* 107.0495  FI at *m/z* 105.0569  FI at *m/z* 91.0549  FI at *m/z* 84.0814 | 58  37  2  2  3  2  100  2  3  66 | 411.1914  305.1495  194.0811  190.1226  174.1277  166.0862  107.0491  105.0573  91.0542  84.0807 | C_23_H_27_O_5_N_2_  C_16_H_21_O_4_N_2_  C_10_H_12_O_3_N  C_12_H_16_ON  C_12_H_16_N  C_9_H_12_O_2_N  C_7_H_7_O  C_7_H_7_N  C_7_H_7_  C_5_H_10_N | -0.49  -0.33  3.09  3.16  2.30  0.60  3.74  -3.81  7.69  8.33 | 4.11 |
| M24 | Epoxidation + hydrolyze + hydroxylation isomer 2 | PI at *m/z* 411.1912  FI at *m/z* 393.1803  FI at *m/z* 267.1853  FI at *m/z* 174.1277  FI at *m/z* 172.1119  FI at *m/z* 91.0549  FI at *m/z* 84.0815  FI at *m/z* 82.0658 | 67  11  5  93  5  100  2  3 | 411.1914  393.1808  267.1855  174.1277  172.1120  91.0542  84.0807  82.0651 | C_23_H_27_O_5_N_2_  C_23_H_25_O_4_N_2_  C_18_H_23_N_2_  C_12_H_16_N  C_12_H_14_N  C_7_H_7_  C_5_H_10_N  C_5_H_8_N | -0.49  -1.27  -0.75  0.00  -0.58  7.69  9.51  8.53 | 4.16 |
| M25 | Epoxidation + hydrolyze + hydroxylation isomer 3 | PI at *m/z* 411.1910  FI at *m/z* 305.1493  FI at *m/z* 287.1392  FI at *m/z* 194.0813  FI at *m/z* 190.1225  FI at *m/z* 166.0863  FI at *m/z* 107.0495  FI at *m/z* 84.0815 | 100  19  2  3  16  3  71  88 | 411.1914  305.1495  287.1390  194.0811  190.1226  166.0862  107.0491  84.0807 | C_23_H_27_O_5_N_2_  C_16_H_21_O_4_N_2_  C_16_H_19_O_3_N_2_  C_10_H_12_O_3_N  C_12_H_16_ON  C_9_H_12_O_2_N  C_7_H_7_O  C_5_H_10_N | -0.97  -0.66  0.70  1.03  -0.53  0.60  3.74  9.51 | 4.54 |
| M26 | Dihydroxylation + sulfation | PI at *m/z* 473.1366  FI at *m/z* 393.1804  FI at *m/z* 286.0742  FI at *m/z* 271.1441  FI at *m/z* 206.1176  FI at *m/z* 203.0008  FI at *m/z* 188.0706  FI at *m/z* 178.0863  FI at *m/z* 123.0443  FI at *m/z* 95.0132  FI at *m/z* 84.0814 | 70  10  9  76  3  43  11  13  81  8  100 | 473.1376  393.1808  286.0743  271.1441  206.1175  203.0008  188.0706  178.0862  123.0440  95.0127  84.0807 | C_23_H_27_O_7_N_2_S  C_23_H_25_O_4_N_2_  C_12_H_16_O_5_NS  C_16_H_19_O_2_N_2_  C_12_H_16_O_2_N  C_7_H_7_O_5_S  C_11_H_10_O_2_N  C_10_H_12_O_2_N  C_7_H_7_O_2_  C_5_H_3_O_2_  C_5_H_10_N | -2.11  -1.02  -0.35  0.00  0.49  0.00  0.00  0.56  2.44  5.26  8.33 | 5.10 |
| M27 | Hydroxylation + glucuronidation | PI at *m/z* 553.2186  FI at *m/z* 377.1861  FI at *m/z* 271.1441  FI at *m/z* 188.0705  FI at *m/z* 107.0495  FI at *m/z* 84.0814 | 26  2  14  2  100  13 | 553.2180  377.1859  271.1441  188.0706  107.0491  84.0807 | C_29_H_32_O_9_N_2_  C_23_H_25_O_3_N_2_  C_16_H_19_O_2_N_2_  C_11_H_10_O_2_N C_7_H_7_O  C_5_H_10_N | 1.08  0.53  0.00  -0.53  3.74  8.33 | 4.31 |

**Table S3** Isozyme mapping of the identified 4F-Cy-BAP and Fu-BAP metabolites in comparison to pooled human liver S9 fraction (pHLS9) and pooled human liver microsomes (pHLM) incubations. Metabolite IDs correspond to Table S2. Cytochrome P450 (CYP); flavin-containing monooxygenase (FMO); **+**, detected; - not detected, *considered to be artifacts.

| *Parent compound*  Metabolite ID | pHLS9 | pHLM | CYP | | | | | | | | | | FMO | | |
| --- | --- | --- | --- | --- | --- | --- | --- | --- | --- | --- | --- | --- | --- | --- | --- |
|  |  |  | 1A2 | 2A6 | 2B6 | 2C8 | 2C9 | 2C19 | 2D6 | 2E1 | 3A4 | 3A5 | | 3 |  |
| *4F-Cy-BAP* | | | | | | | | | | | | | | | |
| M1 | **+** | **+** | **+** | **-** | **-** | **-** | **-** | **+** | **-** | **-** | **+** | **+** | | **-** |  |
| M2* | **+** | **+** | **-** | **-** | **-** | **-** | **-** | **-** | **-** | **-** | **-** | **-** | | **-** |  |
| M3* | **+** | **+** | **-** | **-** | **-** | **-** | **-** | **-** | **-** | **-** | **-** | **-** | | **-** |  |
| M5 | **-** | **-** | **-** | **-** | **-** | **-** | **-** | **+** | **+** | **-** | **-** | **-** | | **-** |  |
| M6 | **-** | **-** | **-** | **-** | **-** | **-** | **-** | **+** | **-** | **-** | **-** | **-** | | **-** |  |
| M7 | **+** | **+** | **-** | **-** | **-** | **-** | **-** | **-** | **-** | **-** | **+** | **+** | | **-** |  |
| *Fu-BAP* | | | | | | | | | | | | | | | |
| M9 | **+** | **+** | **-** | **-** | **-** | **-** | **-** | **-** | **-** | **-** | **-** | **-** | | **-** |  |
| M10 | **+** | **+** | **+** | **-** | **-** | **+** | **-** | **+** | **+** | **-** | **+** | **-** | | **-** |  |
| M11 | **+** | **+** | **-** | **-** | **-** | **-** | **-** | **-** | **-** | **-** | **-** | **-** | | **-** |  |
| M12 | **+** | **+** | **-** | **-** | **-** | **-** | **-** | **-** | **-** | **-** | **-** | **-** | | **-** |  |
| M15 | **+** | **+** | **-** | **-** | **-** | **-** | **-** | **+** | **-** | **-** | **+** | **-** | | **-** |  |
| M16 | **-** | **-** | **-** | **-** | **-** | **-** | **-** | **+** | **-** | **-** | **-** | **-** | | **-** |  |
| M17 | **+** | **+** | **-** | **-** | **-** | **+** | **-** | **+** | **-** | **-** | **+** | **-** | | **-** |  |
| M18 | **-** | **+** | **-** | **-** | **-** | **-** | **-** | **+** | **-** | **-** | **-** | **-** | | **-** |  |
| M20 | **+** | **+** | **-** | **-** | **-** | **-** | **-** | **-** | **+** | **-** | **-** | **-** | | **-** |  |
| M22 | **-** | **-** | **-** | **-** | **-** | **-** | **-** | **-** | **+** | **-** | **-** | **-** | | **-** |  |
| M24 | **-** | **-** | **-** | **-** | **-** | **-** | **-** | **-** | **+** | **-** | **+** | **-** | | **-** |  |

**Table S4** EC_50_ (potency) and E_max_ (maximal activity) values (95% CI profile likelihood) of 4F-Cy-BAP and Fu-BAP are given in comparison to the model substrates hydromorphone (HM) and fentanyl.

|  | EC_50_ (95%CI) | E_max_ (95%CI) |
| --- | --- | --- |
| HM | 10.73 nM (7.13-16.2 nM) | 100% (94.6-106%) |
| Fentanyl | 23.4 nM (21.4-25.4 nM) | 180% (176-183%) |
| 4F-Cy-BAP | 3620 nM (2422-7782 nM) | 5.98% (5.20-8.46%) |
| Fu-BAP | >10 µM | 0.98% (0.90-1.06%) |

**Fig. S1** MS^2^ spectra of the lower abundant metabolites of 4F-Cy-BAP identified in all investigated in vitro and in vivo models sorted by precursor ion (PI) and retention time (RT). Metabolite IDs correspond to Table S2. Metabolites marked with an asterisk are most likely artifacts.


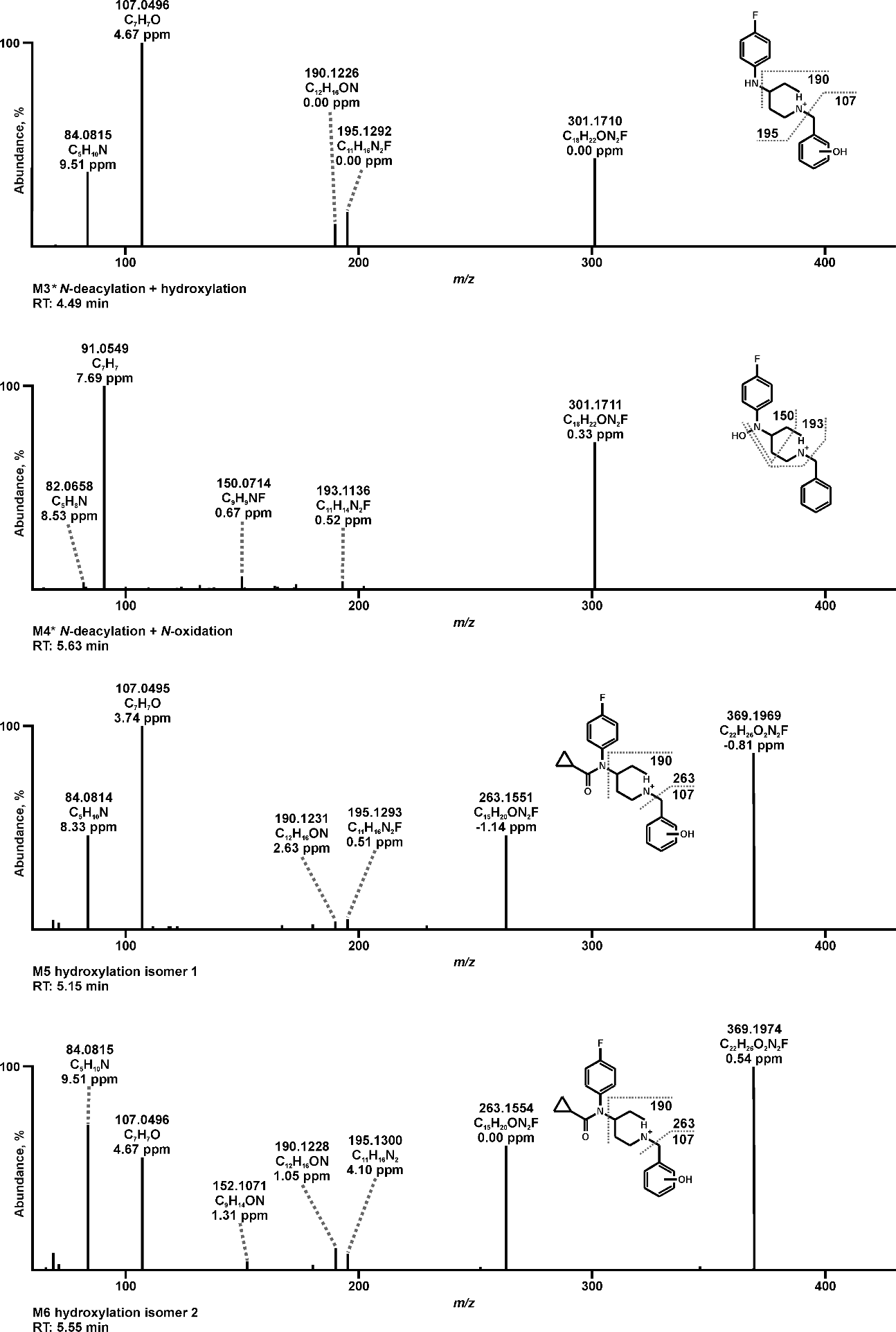


**Fig. S1** (continued)


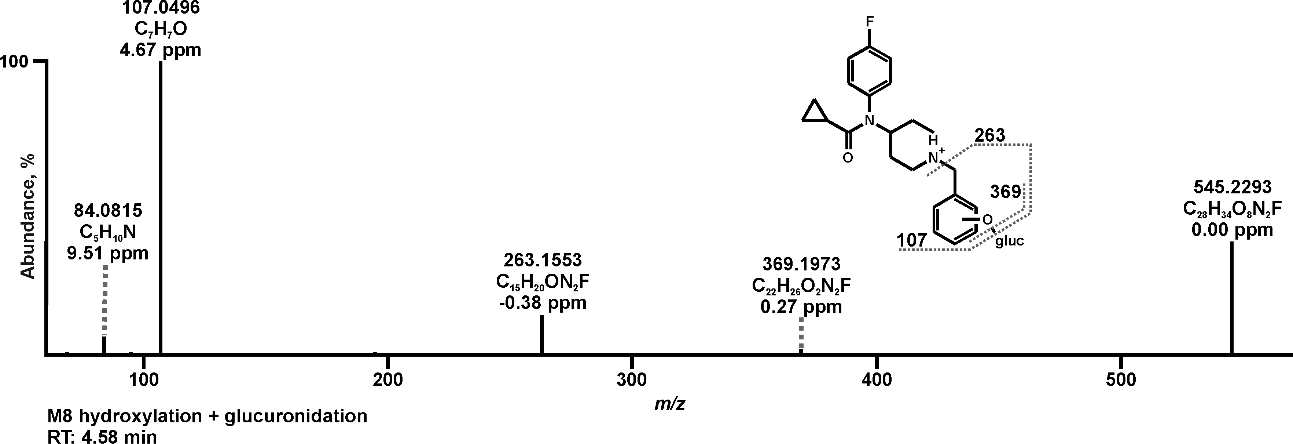


**Fig. S2** MS^2^ spectra of the lower abundant metabolites of Fu-BAP identified in all investigated in vitro and in vivo models sorted by precursor ion (PI) and retention time (RT). Metabolite IDs correspond to Table S2. The metabolite marked with an asterisk is most likely an artifact.


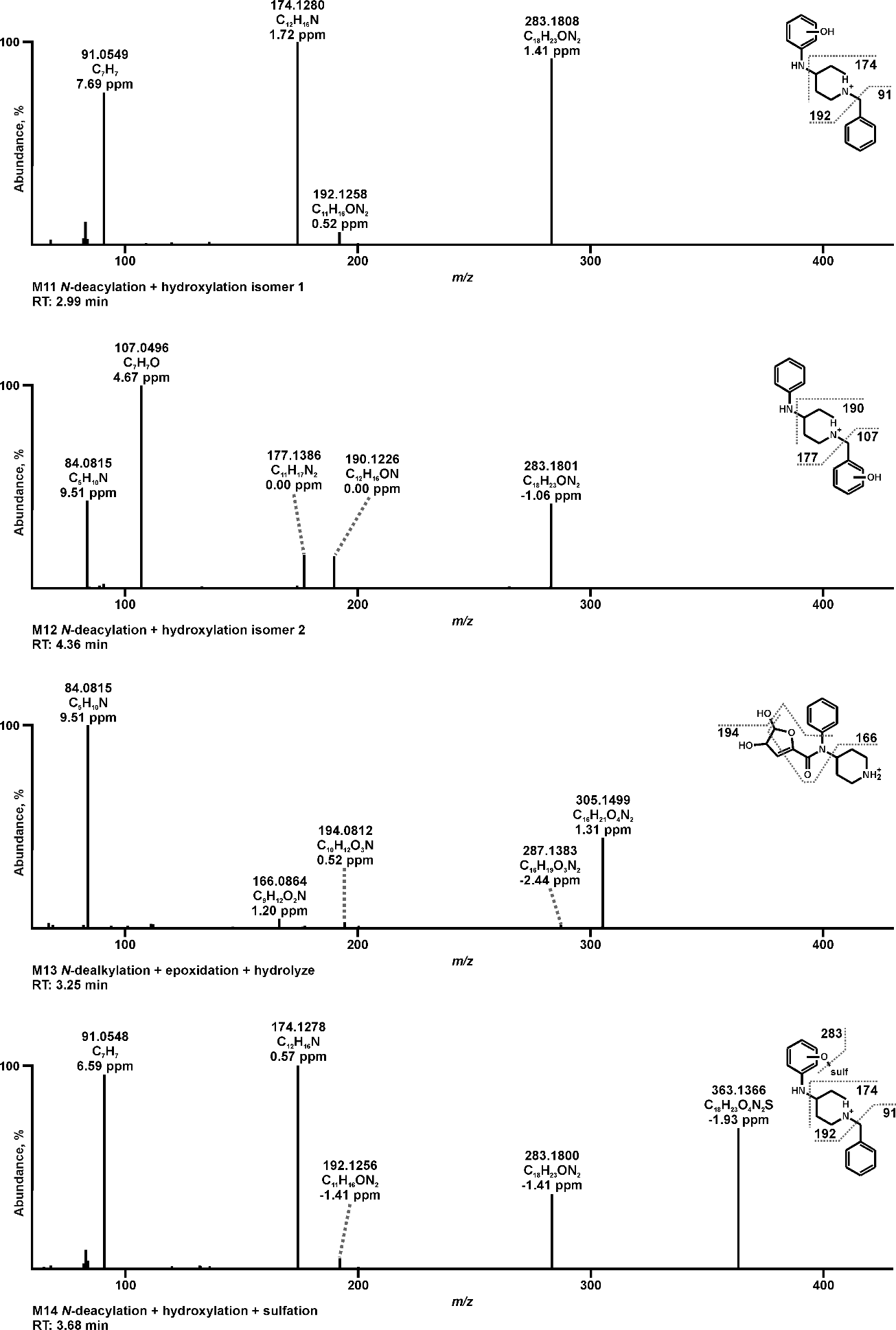


**Fig. S2** (continued)


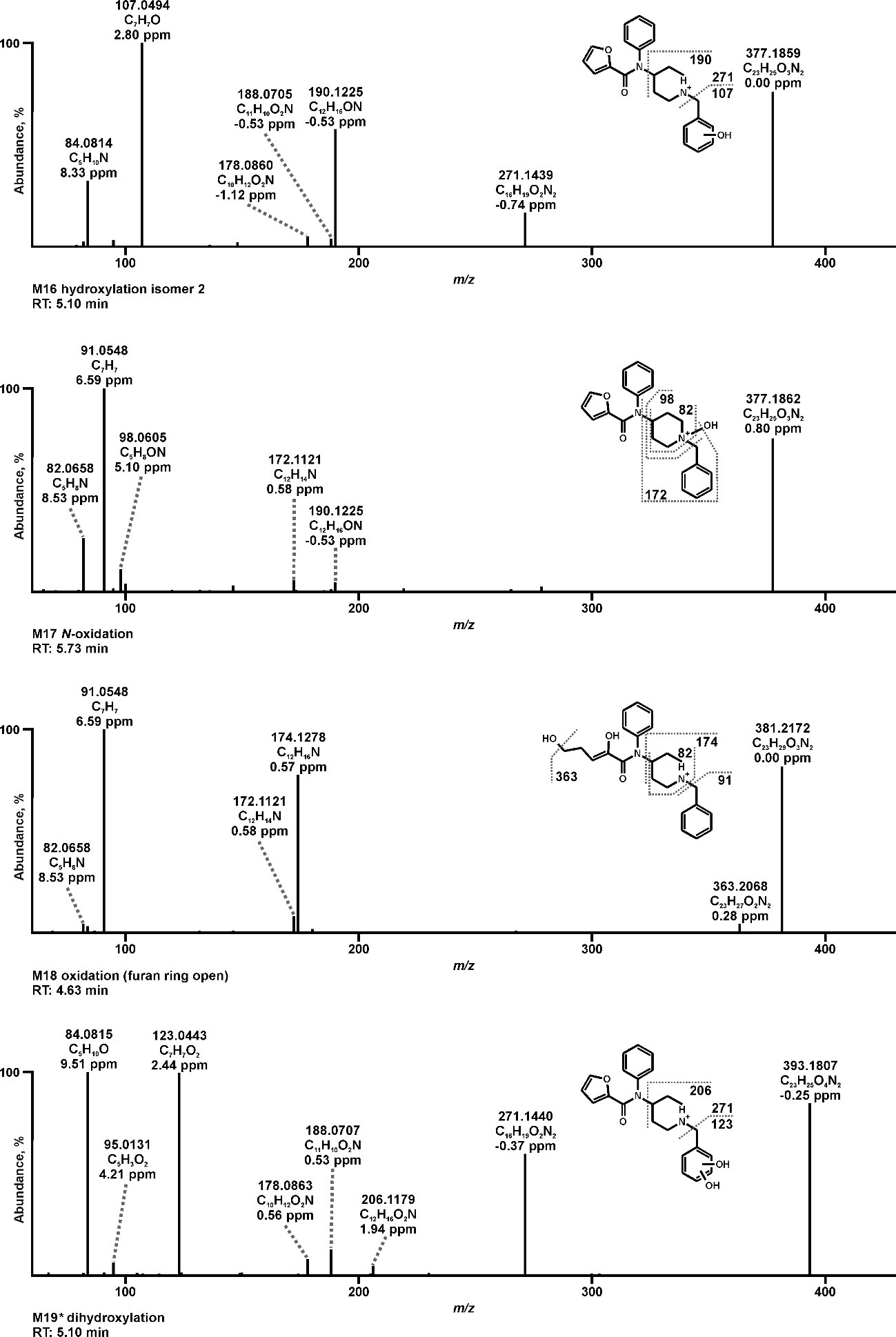


**Fig. S2** (continued)


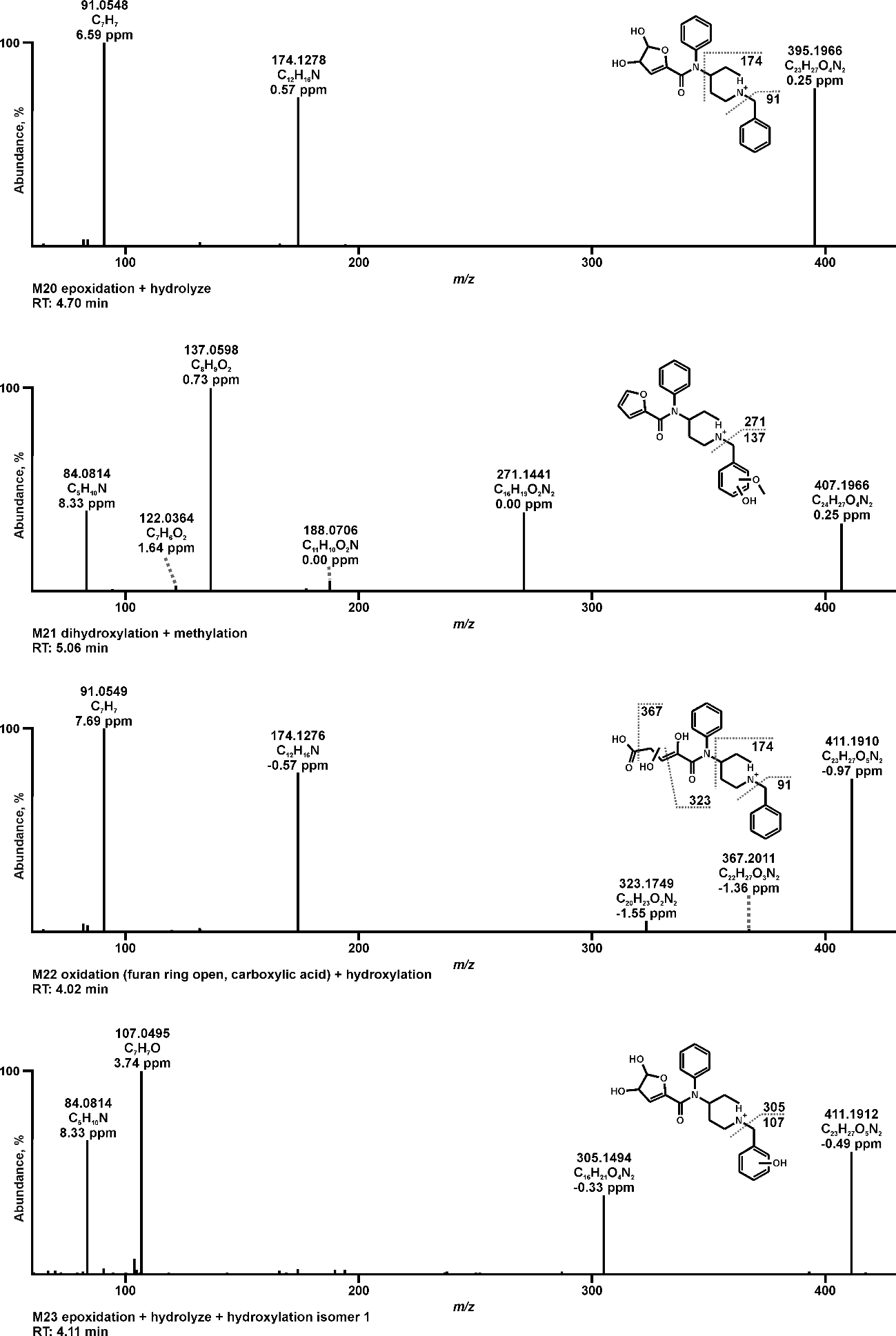


**Fig. S2** (continued)


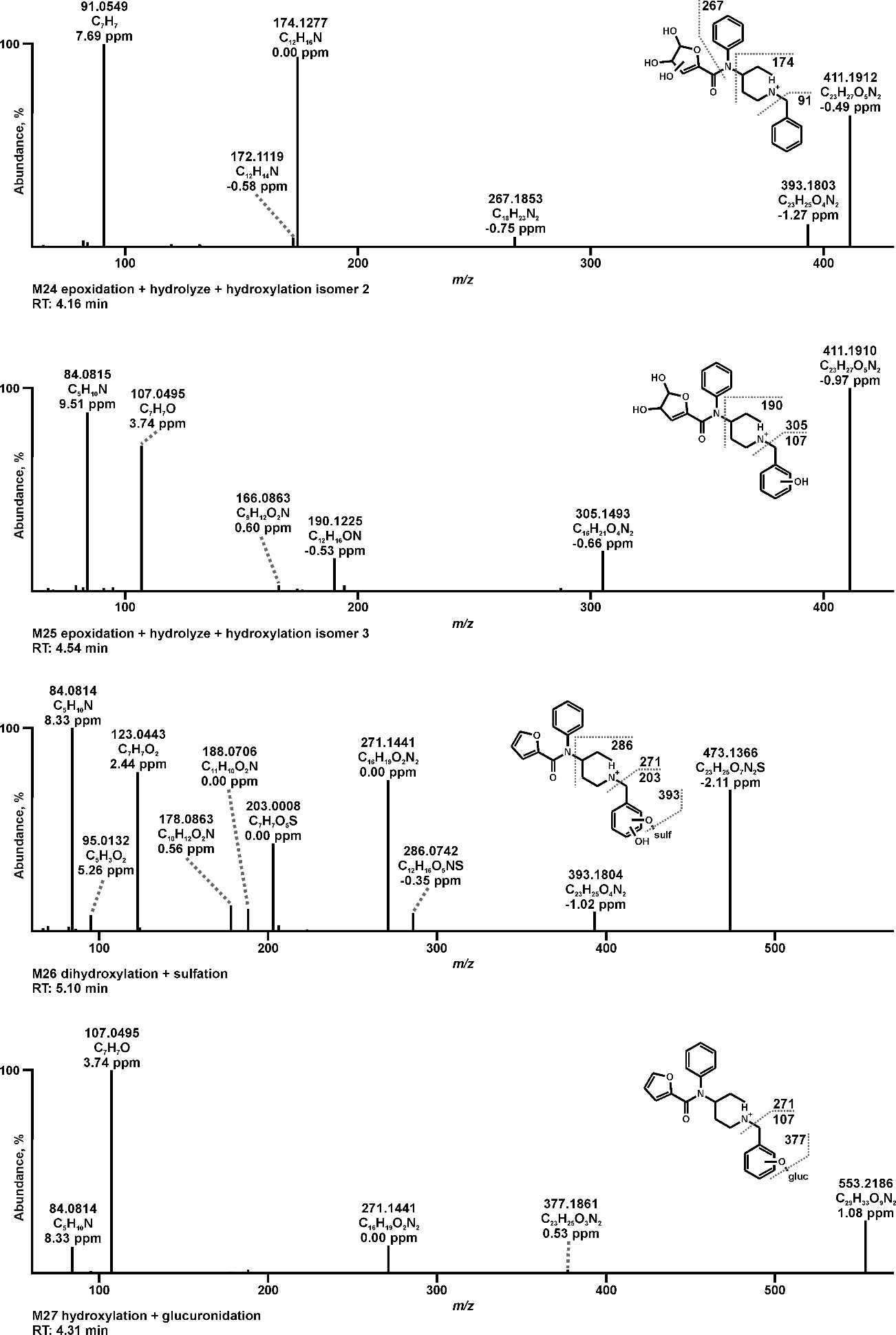

Supplement: Supplementary file 1 — Supplementary file1 (DOCX 1680 kb) [file 204_2020_2726_MOESM1_ESM.docx]
